# Author Correction: Human macrophages differentially produce specific resolvin or leukotriene signals that depend on bacterial pathogenicity

**DOI:** 10.1038/s41467-023-41667-y

**Published:** 2023-09-28

**Authors:** Oliver Werz, Jana Gerstmeier, Stephania Libreros, Xavier De la Rosa, Markus Werner, Paul C. Norris, Nan Chiang, Charles N. Serhan

**Affiliations:** 1 Center for Experimental Therapeutics and Reperfusion Injury, Department of Anesthesia, Perioperative and Pain Medicine, Brigham and Women’s Hospital and Harvard Medical School, 60 Fenwood Road, BTM 3016, Boston, MA 02115 USA; 2 Department of Pharmaceutical/Medicinal Chemistry, Institute of Pharmacy, Friedrich-Schiller-University Jena, Philosophenweg 14, 07743 Jena, Germany

Correction to: *Nature Communications* 10.1038/s41467-017-02538-5, published online 04 January 2018

The original version of this Article contained an error in Fig. 1a, in which the two chromatograms for RvD2 and RvD5 were produced by re-plotting the raw LC–MS–MS output and omitting peaks considered of less relevance because there were no other prominent MRM pecks from this targeted analysis; this illustration of the data is misleading to readers as it does not show the full original chromatograms. This has been corrected in both the PDF and HTML versions of the Article by replacing Fig. 1a with chromatograms obtained from the targeted analysis using direct screen captures as well as those obtained from the standards of RvD2 and RvD5. Both the original and updated versions of Fig. 1a are shown below for reference purposes.

Incorrect Fig. 1a


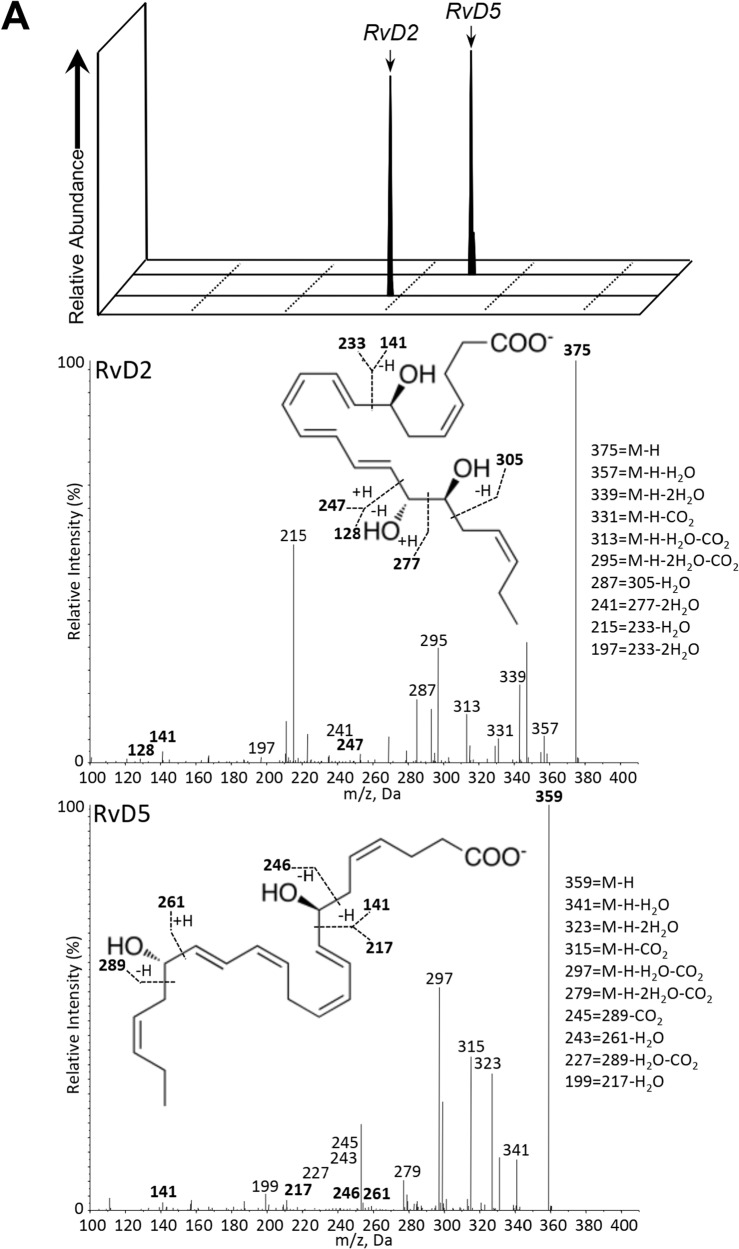


Updated Fig. 1a


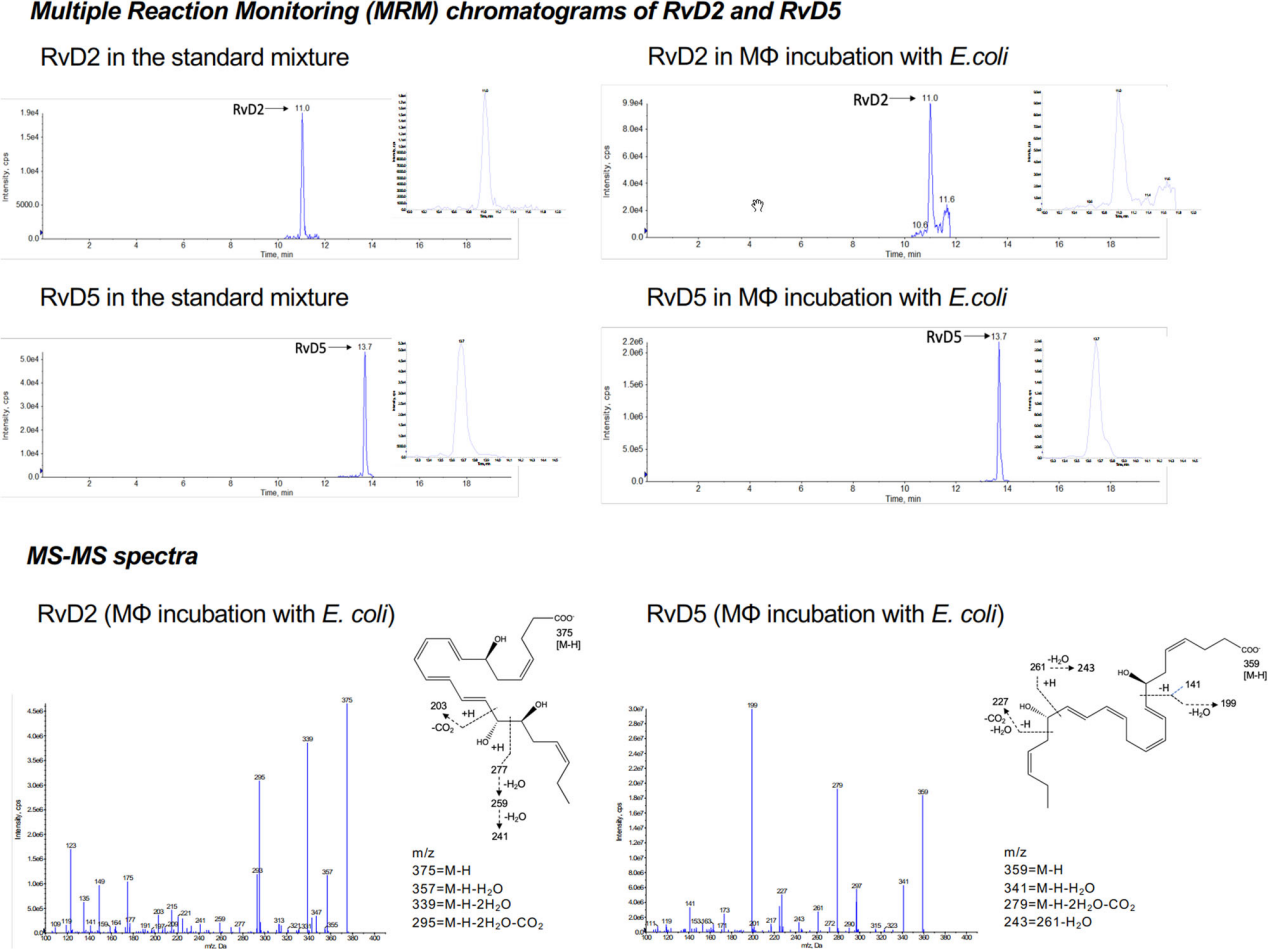


The Source Data file has also been added to include all biological replicates for Fig. 1a and western blot in Supplementary Fig. 3b.

There was also an error in Fig. 3b, in which the CD80 and CD54 plots were inadvertently used twice for both 24 and 48 h time points. This has been corrected in both the PDF and HTML versions of the Article.

### Supplementary information

Updated Supplementary Information

### Source data

Source Data file

