# Supplementary material for: Human macrophages differentially produce specific resolvin or leukotriene signals that depend on bacterial pathogenicity
Source: Nat Commun. 2018 Jan 4;9:59. doi: 10.1038/s41467-017-02538-5 (PMC5754355; doi:10.1038/s41467-017-02538-5)
Supplement: Supplementary file 1 — Supplementary Information [file 41467_2017_2538_MOESM1_ESM.pdf]

Supplementary Figure 1

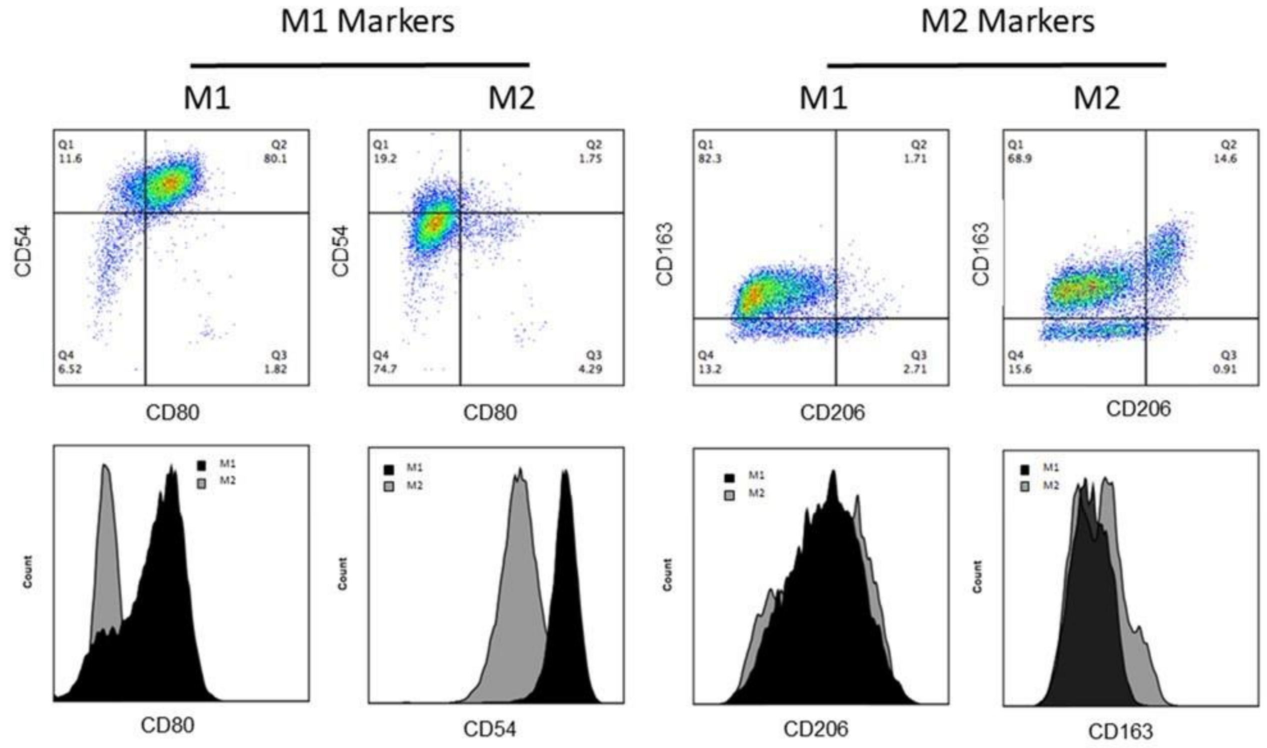

Supplementary Fig. 1. Assessment of macrophage polarization. Human monocytes were differentiated by GM-CSF or M-CSF (20 ng/ml, each) for 6 days to obtain $M0_{GM\text{-}CSF}$ or $M0_{MCSF}$, respectively. Cells were either polarized with 100 ng/ml LPS plus 20 ng/ml IFN-γ to obtain M1, or with 20 ng/ml IL-4 to obtain M2. After 48 h, cells were analyzed for surface expression of polarization markers by flow cytometry. Results are representative for at least three experiments.

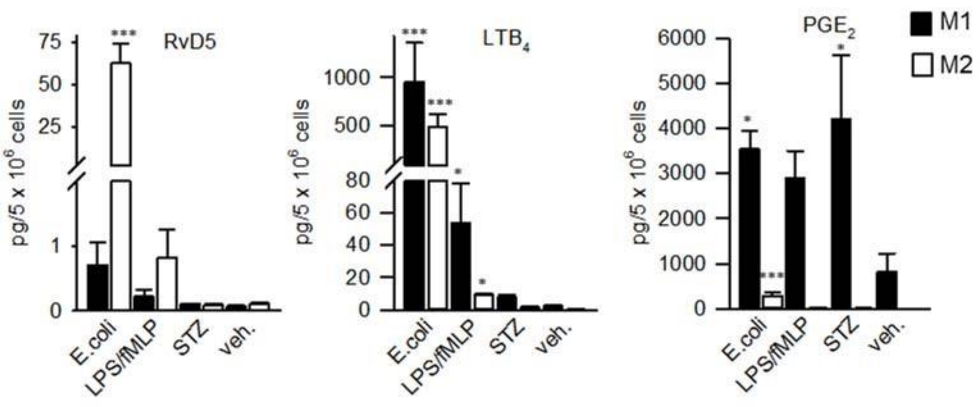

Supplementary Figure 2

Supplementary Fig. 2. Lipid mediator formation in M1 and M2 in response to different stimuli. Human monocyte-derived macrophages were polarized for 24 h to M1 and M2. Cells (5×10^6 cells/ml PBS+Ca/Mg) were incubated at 37 °C for 60 min with *E. coli* (O6:K2:H1; ratio = 1:50), 100 ng/ml LPS plus 100 nM fMLF, 100 ng/ml serum-treated zymosan (STZ) or vehicle-treated (veh). Formed lipid mediators were isolated by SPE and analyzed by LC-MSMS. Data are expressed as means ± S.E.M., $n = 3$; *, $p < 0.05$; ***, $p < 0.001$ versus vehicle control (veh.); data were log-transformed for statistical analysis using one-way ANOVA with Bonferroni Multiple Comparison Test.

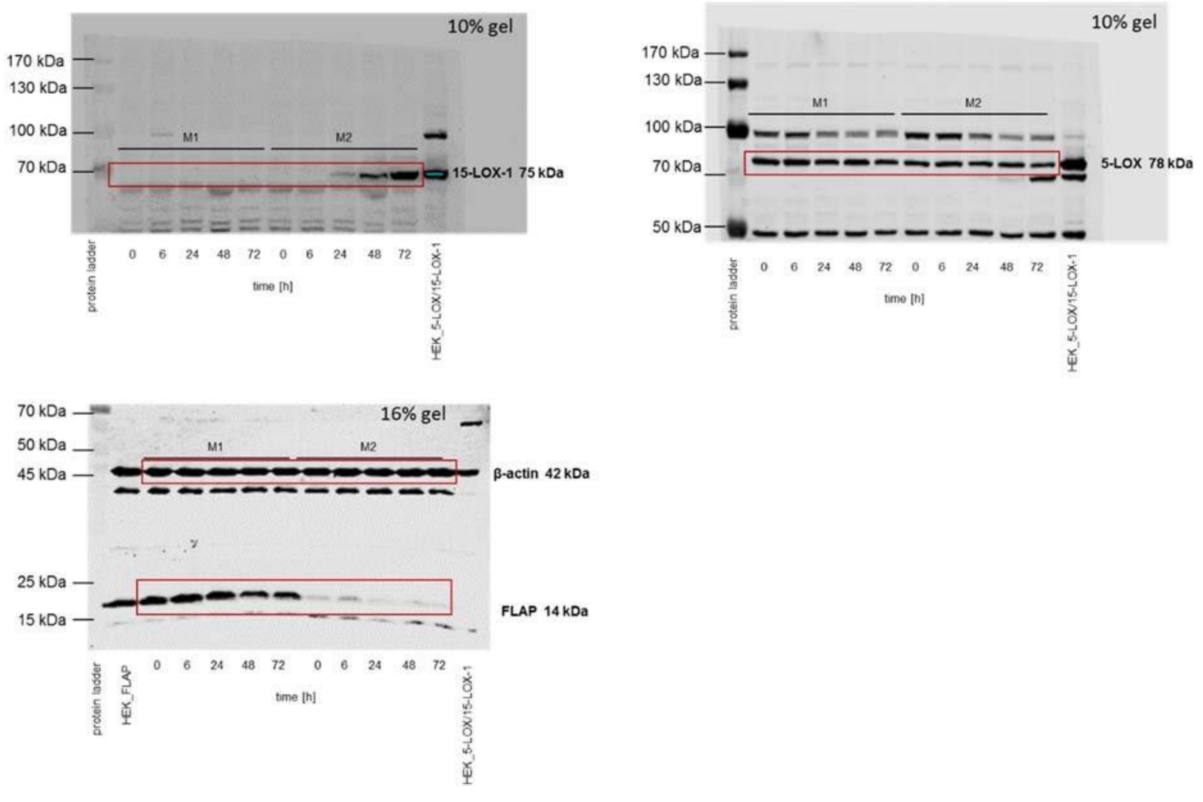

Supplementary Fig. 3. Scans of Western blots shown in Figure 3B. Molecular weight markers are indicated. Red boxes highlight the lanes that are displayed in the corresponding figures.

Supplementary Figure 3

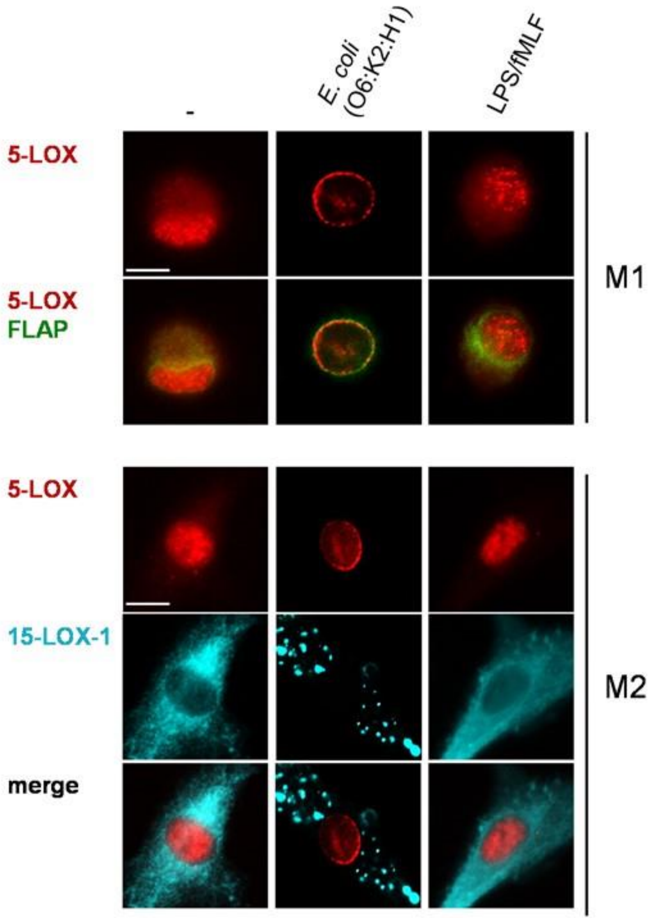

Supplementary Fig. 4. Effects of *E. coli* or LPS/fMLF on the subcellular localization of LOXs. Human M1 or M2 (1×10^6 cells/ml PBS+Ca/Mg) were incubated with or without *E. coli* (O6:K2:H1; ratio = 1:50) for 90 min or with LPS (100 ng/ml) 30 min before further stimulation with fMLF (100 nM, 90 min) at 37 °C. Then, cells were fixed, permeabilized, and incubated with antibodies against 5-LOX (red), FLAP (green) or 15-LOX-1 (cyan-blue); scale bars = 10 µm. Results shown for one single cell are representative for approximately 100 individual cells analyzed in *n* = 3 independent experiments (separate donors), each.

Supplementary Figure 4

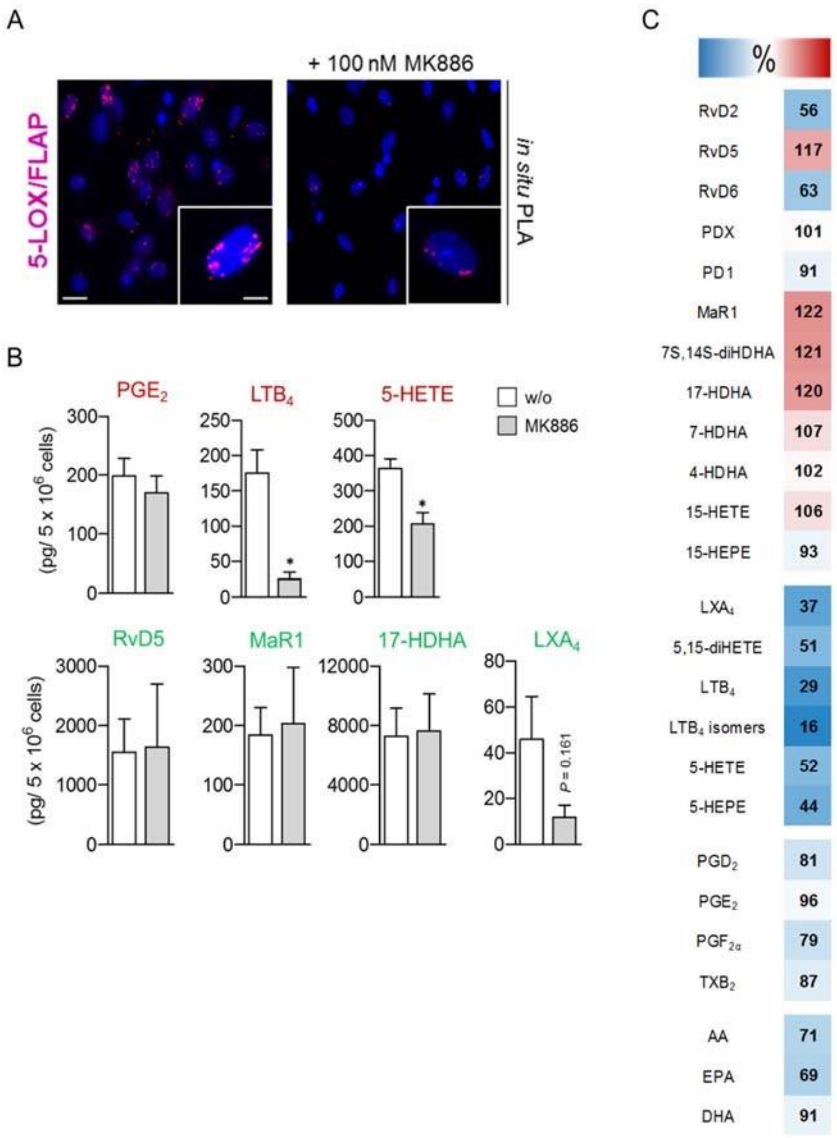

Supplementary Fig. 5. Effects of the FLAP inhibitor MK886 on lipid mediator biosynthesis in M2. (A) Effects of MK886 on the 5-LOX/FLAP complex assembly. M2 (1×10^6 cells/ml PBS+Ca/Mg) were preincubated with 100 nM MK886 or vehicle (0.1% DMSO) for 15 min at 37 °C, and then incubated with *E. coli* (O6:K2:H1; ratio = 1:50) for another 90 min. 5-LOX/FLAP complexes (magenta dots) were analyzed by proximity ligation assay (PLA); DAPI (blue) was used to stain the nucleus; scale bars = 5 µm (insets) and 15 µm (overview). Results shown for one single cell (insets) are representative for approximately 100 individual cells analyzed in *n* = 3 independent experiments (separate donors), each. (B,C) M2

(5×10^6 cells/ml PBS+Ca/Mg) were preincubated with 100 nM MK886 or vehicle (w/o, 0.1% DMSO) for 15 min at 37 °C, and then incubated with *E. coli* (ratio = 1:50) for another 90 min. Formed LM were extracted by SPE and analyzed by LC-MS-MS. (B) Selected LM and effects of MK886 on LM formation. Data are given as means ± S.E.M, $n = 3$. *, $p < 0.05$ versus vehicle control as determined by two-tailed t test. (C) Effects of MK886 on the LM signature profile. Results are given as percentage of vehicle-treated M2 (= 100% control, white) and are shown as heat map, $n = 3$.

Supplementary Figure 6

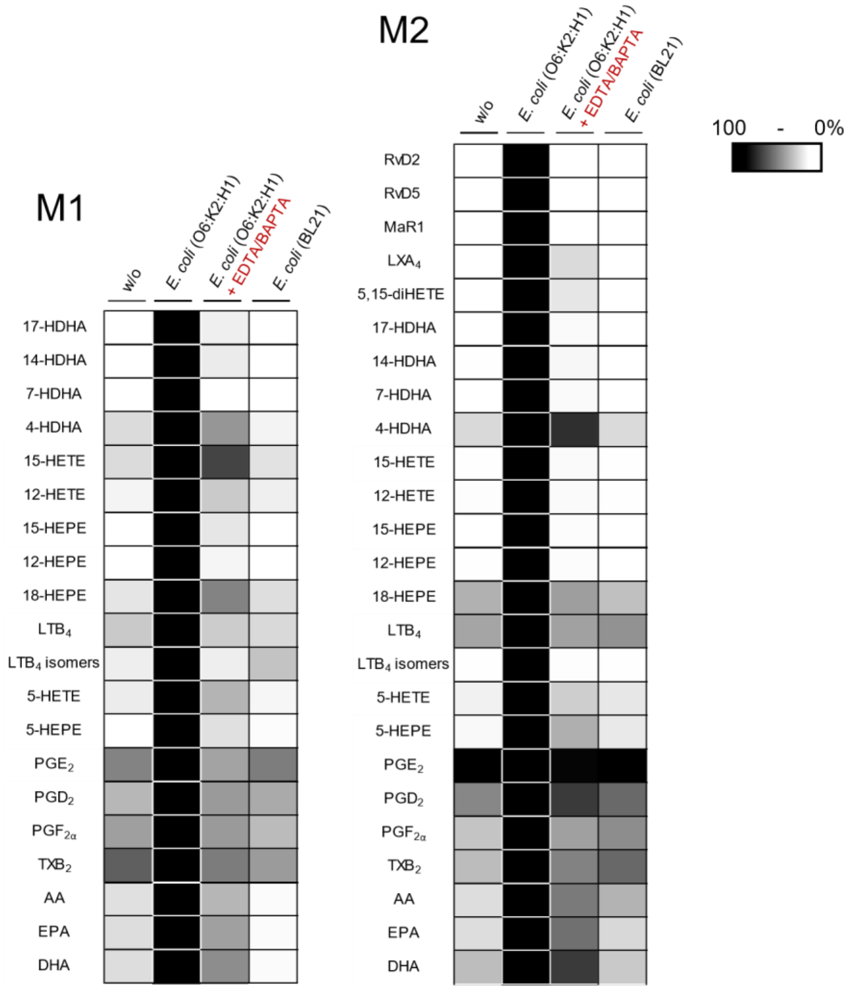

Supplementary Fig. 6. Role of Ca^{2+} and pathogenicity for bacteria-stimulated lipid mediator (LM) formation in M1 and M2. M1 or M2 (5×10^6 cells/ml) were incubated at 37 °C for 90 min with or without *E. coli* (O6:K2:H1; ratio = 1:50) in PBS+Ca/Mg or in PBS containing 0.5 mM EDTA and 20 µM BAPTA/AM, or with the non-pathogenic *E. coli* strain BL21 (ratio = 1:50). Formed LM were isolated by SPE and analyzed by LC-MS/MS. Results are given as percentage of *E. coli* (O6:K2:H1)-stimulated cells treated in PBS+Ca/Mg (= 100% control, black) and shown as heat map, *n* = 3.

Source Data

			M1		M2	
**DHA bioactive metabolome**	**Q1**	**Q3**	**w/o**	***+ E. coli***	**w/o**	***+ E. coli***
RvD1	**375**	**121**	-	-	-	2.8 ± 0.9
RvD2	**375**	**215**	-	1.6 ± 0.5	0.6 ± 0.1	83.2 ± 31.4
RvD5	**359**	**199**	-	4.7 ± 1.4	-	461.9 ± 93.4
RvD6	**359**	**101**	-	0.8 ± 0.4	-	4.5 ± 0.5
AT-RvD1	**375**	**215**	-	1.0 ± 0.4	-	5.4 ± 2.8
PD1	**359**	**153**	-	-	-	7.7 ± 0.7
AT-PD1	**359**	**153**	-	-	-	17.0 ± 2.5
10S,17S-diHDHA	**359**	**153**	-	-	-	53.8 ± 27.5
MaR1	**359**	**221**	-	2.1 ± 0.3	-	84.0 ± 21.7
7S,14S-diHDHA	**359**	**221**	-	0.9 ± 0.3	0.7 ± 0.4	82.0 ± 18.3
4S,14S-diHDHA	**359**	**101**	-	-	-	15.3 ± 4.7
17-HDHA	**343**	**245**	3.1 ± 2.1	80.4 ± 30.7	4.1 ± 2.9	1801.2 ± 627.6
14-HDHA	**343**	**205**	-	32.5 ± 12.4	0.5 ± 0.1	646.2 ± 80.8
7-HDHA	**343**	**141**	-	59.1 ± 28.5	-	141.0 ± 27.9
4-HDHA	**343**	**101**	0.6 ± 0.1	22.5 ± 4.7	1.1 ± 0.4	33.6 ± 7.4
**EPA bioactive metabolome**						
RvE1	**349**	**195**	-	0.5 ± 0.4	-	0.5 ± 0.2
RvE2	**333**	**253**	1.2 ± 0.2	2.4 ± 1.0	3.0 ± 1.5	5.8 ± 2.7
RvE3	**333**	**201**	1.1 ± 0.3	9.7 ± 4.4	2.8 ± 0.8	15.6 ± 7.8
18-HEPE	**317**	**259**	1.0 ± 0.1	7.6 ± 0.6	2.7 ± 0.9	17.0 ± 3.9
15-HEPE	**317**	**219**	-	15.8 ± 3.8	1.5 ± 0.8	1033.9 ± 403.1
12-HEPE	**317**	**179**	-	13.2 ± 4.7	-	147.1 ± 33.8
5-HEPE	**317**	**115**	-	177.5 ± 111.9	0.7 ± 0.2	51.5 ± 18.2

Supplementary Table 1. Biosynthesis of the DHA and EPA metabolome in M1 and M2. Human monocyte-derived macrophages were polarized for 48 h to M1 and M2. Cells (5×10^6 cells/ml PBS+Ca/Mg) were incubated for 90 min with or without (w/o) *E. coli* (O6:K2:H1; ratio = 1:50) at 37 °C. Formed lipid mediators were isolated by SPE and analyzed by LC-MSMS. Lipid mediator profile of M1 and M2; detection limit: 0.5 pg. Data are given as means ± S.E.M., *n* = 7.

Source Data

			M1		M2	
**AA bioactive metabolome**	**Q1**	**Q3**	**w/o**	***+ E. coli***	**w/o**	***+ E. coli***
LXA_4	**351**	**115**	-	3.6 ± 2.0	-	30.2 ± 15.7
AT-LXA_4	**351**	**115**	-	4.4 ± 1.5	-	21.5 ± 4.1
5,15-di-HETE	**335**	**115**	-	32.9 ± 16.7	0.7 ± 0.2	663.4 ± 283.0
LTB_4	**335**	**195**	0.6 ± 0.3	1188.2 ± 639.2	-	239.9 ± 136.9
20-OH-LTB_4	**351**	**195**	-	5.7 ± 2.6	-	3.0 ± 2.1
PGD_2	**351**	**189**	3.9 ± 1.8	172.7 ± 29.2	22.2 ± 12.1	379.2 ± 165.7
PGE_2	**351**	**189**	337.2 ± 242.8	13,525.2 ± 4380.9	15.5 ± 6.7	411.2 ± 106.1
PGF_{2a}	**351**	**193**	66.7 ± 31.8	1116.6 ± 181.6	9.7 ± 4.2	615.8 ± 150.7
TXB_2	**369**	**169**	419.1 ± 220.4	4447.6 ± 1452.1	312.4 ± 148.7	5946.9 ± 863.9
15-HETE	**319**	**219**	1.2 ± 0.2	384.4 ± 96.7	3.3 ± 1.3	3780.3 ± 552.2
12-HETE	**319**	**179**	0.5 ± 0.2	97.6 ± 46.4	0.9 ± 0.2	552.8 ± 112.3
5-HETE	**319**	**115**	0.7 ± 0.2	984.4 ± 583.7	1.8 ± 0.2	592.3 ± 245.0

Supplementary Table 2. Biosynthesis of the AA metabolome in M1 and M2. Human monocyte-derived macrophages were polarized for 48 h to M1 and M2. Cells (5×10^6 cells/ml PBS+Ca/Mg) were incubated for 90 min with or without (w/o) *E. coli* (O6:K2:H1; ratio = 1:50) at 37 °C. Formed lipid mediators were isolated by SPE and analyzed by LC-MS-MS. Lipid mediator profile of M1 and M2; detection limit: 0.5 pg. Data are given as means ± S.E.M., n = 7.

Source Data

			M1		M2	
**Substrates/ fatty acids**	**Q1**	**Q3**	**w/o**	***+ E. coli***	**w/o**	***+ E. coli***
AA	**303**	**259**	492 ± 106	12,762 ± 3298	879 ± 171	9039 ± 2493
EPA	**301**	**257**	112 ± 34	2509 ± 271	170 ± 40	3302 ± 781
DHA	**327**	**283**	1782 ± 365	11,233 ± 4024	1651 ± 213	15,632 ± 5473

Supplementary Table 3. Fatty acid substrate release from M1 and M2. Human monocytederived macrophages were polarized for 48 h to M1 and M2. Cells (5 × 10^6 cells/ml PBS+Ca/Mg) were incubated for 90 min with or without (w/o) *E. coli* (O6:K2:H1; ratio = 1:50) at 37 °C. Released fatty acids were isolated by SPE and analyzed by LC-MS-MS. Lipid mediator profile of M1 and M2; detection limit: 0.5 pg. Data are given as means ± S.E.M., *n* = 7.

Source Data

Step-by-step solid phase extraction and LC-MS-MS of lipid mediators:

1. All cell incubations from individual donors are worked up and subject to LC-MSMS immediately following incubations with or without *E. coli*.

2. Transfer the supernatants of cell incubations (0.5 ml) to 1 ml of ice-cold methanol containing the deuterium-labeled internal standards d_8-5S-HETE, d_4-LTB_4, d_5LXA_4, d_5-RvD2, and d_4-PGE_2 (500 pg each) and vortex for 5 sec.

3. Cell supernatants-methanol suspensions are held at −20°C for 60 min for protein precipitation and then centrifuged at 1,200 × g, 4 °C, 10 min.

4. Each methanolic suspension is transferred separately to a borosilicate glass tube and protein pellets are discarded.

5. Samples are placed on ice bath in an ice bucket (VWR, #M16807-2001) while setting up manifold (Waters #WAT200607) and conditioning columns (Biotage #221-0010-B).

6. Set up the vacuum manifold (Waters #WAT200607) and connect it to a vacuum.

7. Turn on and set the vacuum to ~ -10 psi within the manifold. Pre-wash the SPE column with 3 ml methanol twice, allowing the cartridge bed to saturate with methanol, and then elute. Stop the liquid flow just before the meniscus reaches the top of the cartridge packing. Make sure that all eluents drip in ~ 1-2 drops per second during the elution from SPE.

8. Wash the column with 3 ml of distilled water twice. Wait until all cartridges are stopped at the same level before proceeding.

9. To protonate unesterified carboxylate ($R\text{-}COO^-$) containing molecules in order to have them extract into the organic phase, add 9 ml acidified H_2O (pH 3.5, HCl) to each sample. Rapidly check pH to verify acidification at approximately pH 3.5.

10. Under pressure, rapidly load the sample (from step #5; 10 ml total) onto the conditioned C18 SPE column to prevent acid-induced degradation of sensitive molecules.

11. Wash the C-18 SPE with 3 ml H_2O to eliminate and discard high polarity compounds.

12. Next, elute the C-18 SPE with 3 ml hexane twice.

13. To collect the products of interest (eicosanoids, resolvins, etc), place a clean borosilicate tube under each column. Elute with total of 6 ml of methyl formate. This fraction contains eicosanoids, SPMs, monohydroxy-containing products and pathway markers.

14. System blank: separately in a borosilicate tube, aliquot 1 ml of methanol containing the same synthetic deuterium-labeled standard mixture: d_8-5S-HETE, d_4-LTB_4, d_5-LXA_4, d_5-RvD2, and d_4-PGE_2 (500 pg each).

15. Next, all samples are taken to dryness under a gentle stream of nitrogen gas using an evaporation system (TurboVap LV, Biotage) with water bath kept at 37°C.

16. Immediately suspend each sample in 50 µl methanol–water (1:1 vol/vol). Vortex for 30 seconds and transfer 50 µl to a clean 1.7 ml Eppendorf tube. Remove precipitates and solid particles by centrifugation at 5,000 × *g*, 4°C, 5 min. Next, for injections into LC-MS-MS, transfer particle-free-supernatant to an autosampler vial (cat #C4010-88AW, Thermo scientific, Rockwood, TN) containing a glass insert (cat #C4010-630, Thermo Scientific, Langwewehe, Germany).

17. Inject samples (40 µl injections into a 50 µl loop) using LC–MS–MS system that is equipped with a Shimadzu LC-20AD HPLC and a Shimadzu SIL-20AC autoinjector (Shimadzu, Kyoto, Japan), coupled with a QTrap 5500 (AB Sciex, Framingham, MA).

18. An InfinityLab Poroshell 120 EC-C18 column (4.6 × 100 mm × 2.7 µm; Agilent Technologies Inc. Santa Clara, CA) is kept in an oven (ThermaSphere TS-130; Phenomenex, Torrance, CA) maintained at 45°C.

19. Elute analytes with a mobile phase flow rate of 0.5 mL/min starting with methanol–water–acetic acid at 20:80:0.01 (vol/vol/vol) for 0.01 min, then ramped to 50:50:0.01 (vol/vol/vol) over 0.19 min and kept at 50:50:0.01 (vol/vol/vol) for 2 min, then ramped to 80:20:0.01 (vol/vol/vol) over 9 min and kept at 80:20:0.01 (vol/vol/vol) for 3.5 min then ramped to 98:2:0.01 (vol/ vol/vol) over 0.1 min and kept at 98:2:0.01 for 5.4 min, then ramped to 20:80:0.01 over 0.1 min and kept at 20:80:0.01 for 2.9 min.

20. In each experiment, inject and acquire the following: inject standard mixture of lipid mediators (to facilitate matching of each lipid mediators of interest), samples of interest, system blank containing deuterium-labeled standards, and blanks (consisting of methanol flush) in between runs.

Source Data

	Time	Module	Event	Parameter
1	0.01	Controller	Start	
2	0.20	Pumps	Pump B Conc.	50
3	2.00	Pumps	Pump B Conc.	50
4	11.00	Pumps	Pump B Conc.	80
5	14.50	Pumps	Pump B Conc.	80
6	14.60	Pumps	Pump B Conc.	98
7	20.00	Pumps	Pump B Conc.	98
8	20.10	Pumps	Pump B Conc.	20
9	23.00	Controller	Stop	
10				

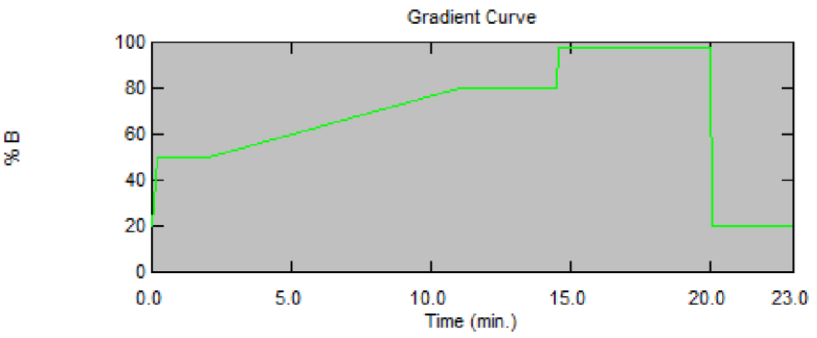

Update Graph

SHIMADZU Solutions for Science

Help

21. AB Sciex QTrap 5500 operated in negative ionization mode using scheduled MRM coupled with information-dependent acquisition (IDA) and enhanced product ions scan. The scheduled MRM window is set at 90 seconds.

Q1 Mass (Da)	***Q3 Mass (Da)***	***RT (min)***	***Param***	***Start***	***Stop***	***ID***
327.3	*116.1*	*17.5*	*CE*	*-17*	*-17*	*d8-5S-HETE*
				CXP	*-10*	*-10*
Q1 Mass (Da)	***Q3 Mass (Da)***	***RT (min)***	***Param***	***Start***	***Stop***	***ID***
339.3	*197.2*	*13.7*	*CE*	*-23*	*-23*	*d4-LTB4*
				CXP	*-13*	*-13*
Q1 Mass (Da)	***Q3 Mass (Da)***	***RT (min)***	***Param***	***Start***	***Stop***	***ID***
355.3	*193.2*	*10.7*	*CE*	*-25*	*-25*	*d4-PGE2*

Source Data

				CXP	*-16*	*-16*
Q1 Mass (Da)	***Q3 Mass (Da)***	***RT (min)***	***Param***	***Start***	***Stop***	***ID***
356.3	*115.2*	*11.5*	*CE*	*-19*	*-19*	*d5-LXA4*
				CXP	*-14*	*-14*
Q1 Mass (Da)	***Q3 Mass (Da)***	***RT (min)***	***Param***	***Start***	***Stop***	***ID***
380.3	*141.2*	*10.9*	*CE*	*-23*	*-23*	*d5-RvD2*
				CXP	*-14*	*-14*
Q1 Mass (Da)	***Q3 Mass (Da)***	***RT (min)***	***Param***	***Start***	***Stop***	***ID***
375.2	*121.1*	*11.3*	*CE*	*-40*	*-40*	*RvD1*
				CXP	*-13*	*-13*
Q1 Mass (Da)	***Q3 Mass (Da)***	***RT (min)***	***Param***	***Start***	***Stop***	***ID***
375.2	*215.1*	*11.3*	*CE*	*-26*	*-26*	*RvD1 2*
				CXP	*-13*	*-13*
Q1 Mass (Da)	***Q3 Mass (Da)***	***RT (min)***	***Param***	***Start***	***Stop***	***ID***
391.1	*231.1*	*10.7*	*CE*	*-24*	*-24*	*22-OH-RvD1/D2*
				CXP	*-13*	*-13*
Q1 Mass (Da)	***Q3 Mass (Da)***	***RT (min)***	***Param***	***Start***	***Stop***	***ID***
405.1	*141.1*	*9.7*	*CE*	*-24*	*-24*	*22-COOH-RvD1/D2*

				CXP	*-13*	*-13*
Q1 Mass (Da)	***Q3 Mass (Da)***	***RT (min)***	***Param***	***Start***	***Stop***	***ID***
373.1	*231.1*	*11.5*	*CE*	*-24*	*-24*	*8/17-oxo-RvD1*
				CXP	*-13*	*-13*
Q1 Mass (Da)	***Q3 Mass (Da)***	***RT (min)***	***Param***	***Start***	***Stop***	***ID***

Source Data

377.1	*141.1*	*11.5*	*CE*	*-24*	*-24*	*9,10/15,16-dihyRvD1+8,9/14,15dihy-RvD2*
				CXP	*-13*	*-13*
Q1 Mass (Da)	***Q3 Mass (Da)***	***RT (min)***	***Param***	***Start***	***Stop***	***ID***
375.2	*141.1*	*11*	*CE*	*-21*	*-21*	*RvD2*
				CXP	*-13*	*-13*
Q1 Mass (Da)	***Q3 Mass (Da)***	***RT (min)***	***Param***	***Start***	***Stop***	***ID***
375.2	*175.1*	*11*	*CE*	*-30*	*-30*	*RvD2 2*
				CXP	*-13*	*-13*
Q1 Mass (Da)	***Q3 Mass (Da)***	***RT (min)***	***Param***	***Start***	***Stop***	***ID***
373.1	*233.1*	*11.5*	*CE*	*-20*	*-20*	*7-oxo-RvD2*
				CXP	*-13*	*-13*
Q1 Mass (Da)	***Q3 Mass (Da)***	***RT (min)***	***Param***	***Start***	***Stop***	***ID***
373.1	*260.1*	*11.5*	*CE*	*-20*	*-20*	*16-oxo-RvD2*
				CXP	*-13*	*-13*
Q1 Mass (Da)	***Q3 Mass (Da)***	***RT (min)***	***Param***	***Start***	***Stop***	***ID***
375.2	*147.1*	*10.9*	*CE*	*-25*	*-25*	*RvD3*
				CXP	*-13*	*-13*
Q1 Mass (Da)	***Q3 Mass (Da)***	***RT (min)***	***Param***	***Start***	***Stop***	***ID***
375.2	*181.1*	*10.9*	*CE*	*-22*	*-22*	*RvD3 3*
				CXP	*-13*	*-13*
Q1 Mass (Da)	***Q3 Mass (Da)***	***RT (min)***	***Param***	***Start***	***Stop***	***ID***

373.1	*163.1*	*11.5*	*CE*	*-22*	*-22*	*oxo-RvD3 1*
				CXP	*-13*	*-13*

Source Data

Q1 Mass (Da)	***Q3 Mass (Da)***	***RT (min)***	***Param***	***Start***	***Stop***	***ID***
373.1	*189.1*	*11.5*	*CE*	*-22*	*-22*	*oxo-RvD3 2*
				CXP	*-13*	*-13*
Q1 Mass (Da)	***Q3 Mass (Da)***	***RT (min)***	***Param***	***Start***	***Stop***	***ID***
375.2	*255.1*	*12.3*	*CE*	*-25*	*-25*	*RvD4*
				CXP	*-18*	*-18*
Q1 Mass (Da)	***Q3 Mass (Da)***	***RT (min)***	***Param***	***Start***	***Stop***	***ID***
375.2	*101.1*	*12.3*	*CE*	*-22*	*-22*	*RvD4 2*
				CXP	*-10*	*-10*
Q1 Mass (Da)	***Q3 Mass (Da)***	***RT (min)***	***Param***	***Start***	***Stop***	***ID***
375.2	*209.1*	*10.8*	*CE*	*-18*	*-18*	*RvT1*
				CXP	*-12*	*-12*
Q1 Mass (Da)	***Q3 Mass (Da)***	***RT (min)***	***Param***	***Start***	***Stop***	***ID***
375.2	*191.1*	*10.8*	*CE*	*-18*	*-18*	*RvT1*
				CXP	*-12*	*-12*
Q1 Mass (Da)	***Q3 Mass (Da)***	***RT (min)***	***Param***	***Start***	***Stop***	***ID***
375.2	*225.1*	*12*	*CE*	*-22*	*-22*	*RvT2+RvD4*
				CXP	*-12*	*-12*
Q1 Mass (Da)	***Q3 Mass (Da)***	***RT (min)***	***Param***	***Start***	***Stop***	***ID***
375.2	*141.11*	*12.2*	*CE*	*-18*	*-18*	*RVT1+2*
				CXP	*-12*	*-12*
Q1 Mass (Da)	***Q3 Mass (Da)***	***RT (min)***	***Param***	***Start***	***Stop***	***ID***
375.2	*171.1*	*12.3*	*CE*	*-18*	*-18*	*RvT3*
				CXP	*-12*	*-12*
Q1 Mass (Da)	***Q3 Mass (Da)***	***RT (min)***	***Param***	***Start***	***Stop***	***ID***
377.21	*143.11*	*11.2*	*CE*	*-21*	*-21*	*7,8,17-triHDPA*
				CXP	*-13*	*-13*

Source Data

Q1 Mass (Da)	Q3 Mass (Da)	RT (min)	Param	Start	Stop	ID
377.2	215	11.2	CE	-26	-26	7,8,17-triHDPA 2
				CXP	-13	-13
**Q1 Mass (Da)**	**Q3 Mass (Da)**	**RT (min)**	**Param**	**Start**	**Stop**	**ID**
377.2	233	10.7	CE	-40	-40	7,16,17-tri-HDPA
				CXP	-13	-13
**Q1 Mass (Da)**	**Q3 Mass (Da)**	**RT (min)**	**Param**	**Start**	**Stop**	**ID**
377.21	261.11	10.7	CE	-28	-28	7,16,17-tri-HDPA 2
				CXP	-13	-13
**Q1 Mass (Da)**	**Q3 Mass (Da)**	**RT (min)**	**Param**	**Start**	**Stop**	**ID**
349.2	195.1	8.6	CE	-22	-22	RvE1
				CXP	-12	-12
**Q1 Mass (Da)**	**Q3 Mass (Da)**	**RT (min)**	**Param**	**Start**	**Stop**	**ID**
349.2	161.1	8.6	CE	-25	-25	RvE1 2
				CXP	-13	-13
**Q1 Mass (Da)**	**Q3 Mass (Da)**	**RT (min)**	**Param**	**Start**	**Stop**	**ID**
347.1	195.1	9	CE	-22	-22	18-oxo-RvE1
				CXP	-10	-10
**Q1 Mass (Da)**	**Q3 Mass (Da)**	**RT (min)**	**Param**	**Start**	**Stop**	**ID**
347.1	289.1	9	CE	-22	-22	12-oxo-RvE1
				CXP	-10	-10
**Q1 Mass (Da)**	**Q3 Mass (Da)**	**RT (min)**	**Param**	**Start**	**Stop**	**ID**
351.2	115.1	11.5	CE	-20	-20	LXA4
				CXP	-13	-13
**Q1 Mass (Da)**	**Q3 Mass (Da)**	**RT (min)**	**Param**	**Start**	**Stop**	**ID**
351.2	235.1	11.5	CE	-20	-20	LXA4 2

Source Data

				CXP	-13	-13
**Q1 Mass (Da)**	**Q3 Mass (Da)**	**RT (min)**	**Param**	**Start**	**Stop**	**ID**
349.1	233.1	11.5	CE	-22	-22	6/15-oxo-LXA4
				CXP	-13	-13
**Q1 Mass (Da)**	**Q3 Mass (Da)**	**RT (min)**	**Param**	**Start**	**Stop**	**ID**
353.1	115.1	11.5	CE	-22	-22	7,8/3,14-dihy-LXA4
				CXP	-13	-13
**Q1 Mass (Da)**	**Q3 Mass (Da)**	**RT (min)**	**Param**	**Start**	**Stop**	**ID**
367.1	115.1	9.8	CE	-22	-22	20-OH-LXA4/B4
				CXP	-13	-13
**Q1 Mass (Da)**	**Q3 Mass (Da)**	**RT (min)**	**Param**	**Start**	**Stop**	**ID**
381.1	115.1	8.8	CE	-22	-22	20-COOH-LXA4/B4
				CXP	-13	-13
**Q1 Mass (Da)**	**Q3 Mass (Da)**	**RT (min)**	**Param**	**Start**	**Stop**	**ID**
349.2	115.1	10.5	CE	-20	-20	LXA5
				CXP	-13	-13
**Q1 Mass (Da)**	**Q3 Mass (Da)**	**RT (min)**	**Param**	**Start**	**Stop**	**ID**
349.2	215.1	10.5	CE	-27	-27	LXA5 2
				CXP	-13	-13
**Q1 Mass (Da)**	**Q3 Mass (Da)**	**RT (min)**	**Param**	**Start**	**Stop**	**ID**
351.2	221.1	11	CE	-20	-20	LXB4
				CXP	-13	-13
**Q1 Mass (Da)**	**Q3 Mass (Da)**	**RT (min)**	**Param**	**Start**	**Stop**	**ID**
351.2	251.1	11	CE	-20	-20	LXB4 2
				CXP	-13	-13
**Q1 Mass (Da)**	**Q3 Mass (Da)**	**RT (min)**	**Param**	**Start**	**Stop**	**ID**

Source Data

349.2	205.2	11	CE	-20	-20	5-oxo-LXB4
				CXP	-11	-11
**Q1 Mass (Da)**	**Q3 Mass (Da)**	**RT (min)**	**Param**	**Start**	**Stop**	**ID**
353.2	223.2	11	CE	-20	-20	6,7-dihy-LXB4
				CXP	-11	-11
**Q1 Mass (Da)**	**Q3 Mass (Da)**	**RT (min)**	**Param**	**Start**	**Stop**	**ID**
349.2	221.1	9.6	CE	-20	-20	LXB5
				CXP	-13	-13
**Q1 Mass (Da)**	**Q3 Mass (Da)**	**RT (min)**	**Param**	**Start**	**Stop**	**ID**
349.2	251.1	9.6	CE	-20	-20	LXB5 2
				CXP	-13	-13
**Q1 Mass (Da)**	**Q3 Mass (Da)**	**RT (min)**	**Param**	**Start**	**Stop**	**ID**
351.3	189.1	10.7	CE	-25	-25	PGE2
				CXP	-14	-14
**Q1 Mass (Da)**	**Q3 Mass (Da)**	**RT (min)**	**Param**	**Start**	**Stop**	**ID**
351.3	175.1	10.7	CE	-25	-25	PGE2 2
				CXP	-14	-14
**Q1 Mass (Da)**	**Q3 Mass (Da)**	**RT (min)**	**Param**	**Start**	**Stop**	**ID**
351.3	233.1	10.8	CE	-16	-16	PGD2
				CXP	-15	-15
**Q1 Mass (Da)**	**Q3 Mass (Da)**	**RT (min)**	**Param**	**Start**	**Stop**	**ID**
353.3	193.1	11	CE	-34	-34	PGF2a
				CXP	-11	-11
**Q1 Mass (Da)**	**Q3 Mass (Da)**	**RT (min)**	**Param**	**Start**	**Stop**	**ID**
369.3	169.1	10.2	CE	-22	-22	TXB2
				CXP	-15	-15
**Q1 Mass (Da)**	**Q3 Mass (Da)**	**RT (min)**	**Param**	**Start**	**Stop**	**ID**

Source Data

351.3	195.1	9	CE	-24	-24	20-OH-LTB4
				CXP	-15	-15
**Q1 Mass (Da)**	**Q3 Mass (Da)**	**RT (min)**	**Param**	**Start**	**Stop**	**ID**

365.3	195.1	8.2	CE	-24	-24	20-COOH-LTB4
				CXP	-15	-15
**Q1 Mass (Da)**	**Q3 Mass (Da)**	**RT (min)**	**Param**	**Start**	**Stop**	**ID**
375.3	153.1	10.1	CE	-24	-24	22-OH-PD1
				CXP	-15	-15
**Q1 Mass (Da)**	**Q3 Mass (Da)**	**RT (min)**	**Param**	**Start**	**Stop**	**ID**
389.3	153.1	9.5	CE	-24	-24	22-COOH-PD1
				CXP	-15	-15
**Q1 Mass (Da)**	**Q3 Mass (Da)**	**RT (min)**	**Param**	**Start**	**Stop**	**ID**
375.3	221.1	10.1	CE	-24	-24	22-OH-MaR1
				CXP	-15	-15
**Q1 Mass (Da)**	**Q3 Mass (Da)**	**RT (min)**	**Param**	**Start**	**Stop**	**ID**
389.3	221.1	9.5	CE	-24	-24	22-COOH-MaR1
				CXP	-15	-15
**Q1 Mass (Da)**	**Q3 Mass (Da)**	**RT (min)**	**Param**	**Start**	**Stop**	**ID**
359.2	153.1	13.1	CE	-21	-21	PD1
				CXP	-9	-9
**Q1 Mass (Da)**	**Q3 Mass (Da)**	**RT (min)**	**Param**	**Start**	**Stop**	**ID**
359.2	181.1	13.1	CE	-19	-19	PD1 2
				CXP	-15	-15
**Q1 Mass (Da)**	**Q3 Mass (Da)**	**RT (min)**	**Param**	**Start**	**Stop**	**ID**

Source Data

357.1	*153.1*	*13.4*	*CE*	*-20*	*-20*	*10/17-oxo-PD1*
				CXP	*-9*	*-9*
Q1 Mass (Da)	***Q3 Mass (Da)***	***RT (min)***	***Param***	***Start***	***Stop***	***ID***
359.2	*231.1*	*14.6*	*CE*	*-20*	*-20*	*16/17-diHDHA*
				CXP	*-16*	*-16*

Q1 Mass (Da)	***Q3 Mass (Da)***	***RT (min)***	***Param***	***Start***	***Stop***	***ID***
359.2	*199.1*	*13.3*	*CE*	*-21*	*-21*	*RvD5*
				CXP	*-13*	*-13*
Q1 Mass (Da)	***Q3 Mass (Da)***	***RT (min)***	***Param***	***Start***	***Stop***	***ID***
359.2	*261.1*	*13.3*	*CE*	*-20*	*-20*	*RvD5 2*
				CXP	*-16*	*-16*
Q1 Mass (Da)	***Q3 Mass (Da)***	***RT (min)***	***Param***	***Start***	***Stop***	***ID***
357.1	*215.2*	*13.5*	*CE*	*-20*	*-20*	*oxoRvD5*
				CXP	*-11*	*-11*
Q1 Mass (Da)	***Q3 Mass (Da)***	***RT (min)***	***Param***	***Start***	***Stop***	***ID***
359.2	*221.1*	*13.5*	*CE*	*-20*	*-20*	*1-Mar*
				CXP	*-16*	*-16*
Q1 Mass (Da)	***Q3 Mass (Da)***	***RT (min)***	***Param***	***Start***	***Stop***	***ID***
359.2	*250.1*	*13.5*	*CE*	*-20*	*-20*	*MaR1 2*
				CXP	*-16*	*-16*
Q1 Mass (Da)	***Q3 Mass (Da)***	***RT (min)***	***Param***	***Start***	***Stop***	***ID***
359.2	*141.1*	*13.5*	*CE*	*-20*	*-20*	*MaR1 3*
				CXP	*-16*	*-16*
Q1 Mass (Da)	***Q3 Mass (Da)***	***RT (min)***	***Param***	***Start***	***Stop***	***ID***
357.2	*219.2*	*13.9*	*CE*	*-20*	*-20*	*7-oxo-MaR1*
				CXP	*-11*	*-11*
Q1 Mass (Da)	***Q3 Mass (Da)***	***RT (min)***	***Param***	***Start***	***Stop***	***ID***

Source Data

357.2	*248.2*	*13.9*	*CE*	*-20*	*-20*	*14-oxo-MaR1*
				CXP	*-11*	*-11*
Q1 Mass (Da)	***Q3 Mass (Da)***	***RT (min)***	***Param***	***Start***	***Stop***	***ID***
359.2	*101.1*	*14*	*CE*	*-22*	*-22*	*RvD6/4,14 1*
				CXP	*-16*	*-16*
Q1 Mass (Da)	***Q3 Mass (Da)***	***RT (min)***	***Param***	***Start***	***Stop***	***ID***

359.2	*159.1*	*14*	*CE*	*-22*	*-22*	*RvD6/4,14 2*
				CXP	*-16*	*-16*
Q1 Mass (Da)	***Q3 Mass (Da)***	***RT (min)***	***Param***	***Start***	***Stop***	***ID***
359.2	*209.1*	*13.9*	*CE*	*-20*	*-20*	*RvT4*
				CXP	*-11*	*-11*
Q1 Mass (Da)	***Q3 Mass (Da)***	***RT (min)***	***Param***	***Start***	***Stop***	***ID***
359.21	*221.11*	*13.9*	*CE*	*-20*	*-20*	*RvT4*
				CXP	*-11*	*-11*
Q1 Mass (Da)	***Q3 Mass (Da)***	***RT (min)***	***Param***	***Start***	***Stop***	***ID***
377.2	*143.1*	*11.2*	*CE*	*-20*	*-20*	*RvD1 n3 DPA 1*
				CXP	*-13*	*-13*
Q1 Mass (Da)	***Q3 Mass (Da)***	***RT (min)***	***Param***	***Start***	***Stop***	***ID***
377.2	*215.1*	*11.2*	*CE*	*-20*	*-20*	*RvD1 n3 DPA 2*
				CXP	*-13*	*-13*
Q1 Mass (Da)	***Q3 Mass (Da)***	***RT (min)***	***Param***	***Start***	***Stop***	***ID***
377.2	*233.1*	*10.8*	*CE*	*-20*	*-20*	*RvD2 n3 DPA 1*
				CXP	*-13*	*-13*

Source Data

Q1 Mass (Da)	Q3 Mass (Da)	RT (min)	Param	Start	Stop	ID
377.2	261.1	10.8	CE	-20	-20	RvD2 n3 DPA 2
				CXP	-13	-13
**Q1 Mass (Da)**	**Q3 Mass (Da)**	**RT (min)**	**Param**	**Start**	**Stop**	**ID**
377.2	211.1	11.2	CE	-20	-20	RvT1 n3 DPA
				CXP	-13	-13
**Q1 Mass (Da)**	**Q3 Mass (Da)**	**RT (min)**	**Param**	**Start**	**Stop**	**ID**
377.2	239.1	11.2	CE	-20	-20	RvT1 n3 DPA 2
				CXP	-13	-13

Q1 Mass (Da)	Q3 Mass (Da)	RT (min)	Param	Start	Stop	ID
377.2	197.1	12.3	CE	-20	-20	RvT2+3 n3 DPA
				CXP	-13	-13
**Q1 Mass (Da)**	**Q3 Mass (Da)**	**RT (min)**	**Param**	**Start**	**Stop**	**ID**
377.2	143.11	12.5	CE	-20	-20	RvT2+3 DPA 2
				CXP	-13	-13
**Q1 Mass (Da)**	**Q3 Mass (Da)**	**RT (min)**	**Param**	**Start**	**Stop**	**ID**
361.1	143.1	14	CE	-20	-20	RvD5 n3 DPA 1
				CXP	-10	-10
**Q1 Mass (Da)**	**Q3 Mass (Da)**	**RT (min)**	**Param**	**Start**	**Stop**	**ID**
361.1	201.2	14	CE	-20	-20	RvD5 n3 DPA 2 +8,9/15,16-dihyRvD5
				CXP	-13	-13

Source Data

Q1 Mass (Da)	Q3 Mass (Da)	RT (min)	Param	Start	Stop	ID
361.1	211.1	14.3	CE	-20	-20	RvT4 n3 DPA
				CXP	-12	-12
**Q1 Mass (Da)**	**Q3 Mass (Da)**	**RT (min)**	**Param**	**Start**	**Stop**	**ID**
361.1	239.1	14.3	CE	-20	-20	RvT4 n3 DPA 2
				CXP	-12	-12
**Q1 Mass (Da)**	**Q3 Mass (Da)**	**RT (min)**	**Param**	**Start**	**Stop**	**ID**
361.1	223.2	14.2	CE	-20	-20	MaR1 n3 DPA 1 +8,9/12,13-dihyMaR1
				CXP	-15	-15
**Q1 Mass (Da)**	**Q3 Mass (Da)**	**RT (min)**	**Param**	**Start**	**Stop**	**ID**

361.1	252.2	14.2	CE	-20	-20	MAR1 n3 DPA 2 +8,9/12,13-dihyMaR1
				CXP	-15	-15
**Q1 Mass (Da)**	**Q3 Mass (Da)**	**RT (min)**	**Param**	**Start**	**Stop**	**ID**
361.1	123.1	14.2	CE	-20	-20	PD1 n3 DPA 1
				CXP	-10	-10
**Q1 Mass (Da)**	**Q3 Mass (Da)**	**RT (min)**	**Param**	**Start**	**Stop**	**ID**
361.1	155.2	14.2	CE	-25	-25	PD1 n3 DPA 2
				CXP	-10.5	-10.5
**Q1 Mass (Da)**	**Q3 Mass (Da)**	**RT (min)**	**Param**	**Start**	**Stop**	**ID**

Source Data

359.2	*205.1*	*13.8*	*CE*	*-22*	*-22*	*14,21-diHDHA*
				CXP	*-16*	*-16*
Q1 Mass (Da)	***Q3 Mass (Da)***	***RT (min)***	***Param***	***Start***	***Stop***	***ID***
359.2	*233.1*	*13.8*	*CE*	*-22*	*-22*	*14,21-diHDHA 2*
				CXP	*-16*	*-16*
Q1 Mass (Da)	***Q3 Mass (Da)***	***RT (min)***	***Param***	***Start***	***Stop***	***ID***
359.2	*231.2*	*14.2*	*CE*	*-22*	*-22*	*16,17-diHDHA*
				CXP	*-16*	*-16*
Q1 Mass (Da)	***Q3 Mass (Da)***	***RT (min)***	***Param***	***Start***	***Stop***	***ID***
359.2	*191.1*	*14.2*	*CE*	*-22*	*-22*	*13,14-diHDHA*
				CXP	*-16*	*-16*
Q1 Mass (Da)	***Q3 Mass (Da)***	***RT (min)***	***Param***	***Start***	***Stop***	***ID***
361.2	*155.1*	*13.7*	*CE*	*-25*	*-25*	*10,17-diHDPA*
				CXP	*-12*	*-12*

Q1 Mass (Da)	***Q3 Mass (Da)***	***RT (min)***	***Param***	***Start***	***Stop***	***ID***
361.2	*183.1*	*13.7*	*CE*	*-23*	*-23*	*10,17-diHDPA 2*
				CXP	*-12*	*-12*
Q1 Mass (Da)	***Q3 Mass (Da)***	***RT (min)***	***Param***	***Start***	***Stop***	***ID***
361.2	*199.1*	*13.7*	*CE*	*-26*	*-26*	*7,17-diHDPA*
				CXP	*-13*	*-13*
Q1 Mass (Da)	***Q3 Mass (Da)***	***RT (min)***	***Param***	***Start***	***Stop***	***ID***
361.2	*263.1*	*13.7*	*CE*	*-21*	*-21*	*7,17-diHDPA 2*
				CXP	*-14*	*-14*
Q1 Mass (Da)	***Q3 Mass (Da)***	***RT (min)***	***Param***	***Start***	***Stop***	***ID***

Source Data

361.2	*223.1*	*13.7*	*CE*	*-23*	*-23*	*7,14-diHDPA*
				CXP	*-14*	*-14*
Q1 Mass (Da)	***Q3 Mass (Da)***	***RT (min)***	***Param***	***Start***	***Stop***	***ID***
361.2	*252.1*	*13.7*	*CE*	*-19*	*-19*	*7,14-diHDPA 2*
				CXP	*-14*	*-14*
Q1 Mass (Da)	***Q3 Mass (Da)***	***RT (min)***	***Param***	***Start***	***Stop***	***ID***
361.2	*207.1*	*13.8*	*CE*	*-22*	*-22*	*14,21-diHDPA*
				CXP	*-16*	*-16*
Q1 Mass (Da)	***Q3 Mass (Da)***	***RT (min)***	***Param***	***Start***	***Stop***	***ID***
361.2	*235.1*	*13.8*	*CE*	*-22*	*-22*	*14,21-diHDPA*
				CXP	*-16*	*-16*
Q1 Mass (Da)	***Q3 Mass (Da)***	***RT (min)***	***Param***	***Start***	***Stop***	***ID***
361.2	*233.1*	*14.2*	*CE*	*-22*	*-22*	*16,17-diHDPA*
				CXP	*-16*	*-16*

Q1 Mass (Da)	***Q3 Mass (Da)***	***RT (min)***	***Param***	***Start***	***Stop***	***ID***
361.2	*193.2*	*14.2*	*CE*	*-22*	*-22*	*13,14-diHDPA*
				CXP	*-16*	*-16*
Q1 Mass (Da)	***Q3 Mass (Da)***	***RT (min)***	***Param***	***Start***	***Stop***	***ID***
333.3	*199.1*	*12.1*	*CE*	*-24*	*-24*	*RvE2/ 5,15*
				CXP	*-17*	*-17*
Q1 Mass (Da)	***Q3 Mass (Da)***	***RT (min)***	***Param***	***Start***	***Stop***	***ID***
333.3	*253.1*	*12.1*	*CE*	*-20*	*-20*	*RvE2*
				CXP	*-12*	*-12*
Q1 Mass (Da)	***Q3 Mass (Da)***	***RT (min)***	***Param***	***Start***	***Stop***	***ID***

Source Data

333.3	*201.2*	*13.5*	*CE*	*-20*	*-20*	*RvE3*
				CXP	*-12*	*-12*
Q1 Mass (Da)	***Q3 Mass (Da)***	***RT (min)***	***Param***	***Start***	***Stop***	***ID***
333.3	*245.1*	*13.5*	*CE*	*-16*	*-16*	*RvE3*
				CXP	*-14*	*-14*
Q1 Mass (Da)	***Q3 Mass (Da)***	***RT (min)***	***Param***	***Start***	***Stop***	***ID***
335.2	*195.1*	*13.7*	*CE*	*-22*	*-22*	*LTB4*
				CXP	*-13*	*-13*
Q1 Mass (Da)	***Q3 Mass (Da)***	***RT (min)***	***Param***	***Start***	***Stop***	***ID***
335.2	*115.1*	*15.7*	*CE*	*-20*	*-20*	*5S,6R-diHETE*
				CXP	*-13*	*-13*
Q1 Mass (Da)	***Q3 Mass (Da)***	***RT (min)***	***Param***	***Start***	***Stop***	***ID***
335.3	*115.1*	*13.4*	*CE*	*-22*	*-22*	*5,15-diHETE*
				CXP	*-13*	*-13*
Q1 Mass (Da)	***Q3 Mass (Da)***	***RT (min)***	***Param***	***Start***	***Stop***	***ID***
335.3	*235.1*	*13.4*	*CE*	*-22*	*-22*	*5,15-diHETE 2*
				CXP	*-13*	*-13*

Q1 Mass (Da)	***Q3 Mass (Da)***	***RT (min)***	***Param***	***Start***	***Stop***	***ID***
333.3	*115.1*	*12.4*	*CE*	*-22*	*-22*	*5,15-diHEPE*
				CXP	*-13*	*-13*
Q1 Mass (Da)	***Q3 Mass (Da)***	***RT (min)***	***Param***	***Start***	***Stop***	***ID***
333.3	*235.1*	*12.4*	*CE*	*-22*	*-22*	*5,15-diHEPE 2*
				CXP	*-13*	*-13*
Q1 Mass (Da)	***Q3 Mass (Da)***	***RT (min)***	***Param***	***Start***	***Stop***	***ID***
343.2	*255.1*	*17.3*	*CE*	*-17*	*-17*	*21-HDHA*

Source Data

				CXP	*-14*	*-14*
Q1 Mass (Da)	***Q3 Mass (Da)***	***RT (min)***	***Param***	***Start***	***Stop***	***ID***
343.2	*245.1*	*17.3*	*CE*	*-17*	*-17*	*17-HDHA*
				CXP	*-14*	*-14*
Q1 Mass (Da)	***Q3 Mass (Da)***	***RT (min)***	***Param***	***Start***	***Stop***	***ID***
343.2	*205.1*	*17.3*	*CE*	*-17*	*-17*	*14-HDHA*
				CXP	*-14*	*-14*
Q1 Mass (Da)	***Q3 Mass (Da)***	***RT (min)***	***Param***	***Start***	***Stop***	***ID***
343.2	*193.1*	*17.3*	*CE*	*-17*	*-17*	*13-HDHA*
				CXP	*-14*	*-14*
Q1 Mass (Da)	***Q3 Mass (Da)***	***RT (min)***	***Param***	***Start***	***Stop***	***ID***
343.2	*141.1*	*17.3*	*CE*	*-18*	*-18*	*7-HDHA*
				CXP	*-15*	*-15*
Q1 Mass (Da)	***Q3 Mass (Da)***	***RT (min)***	***Param***	***Start***	***Stop***	***ID***
343.2	*101.1*	*17.4*	*CE*	*-17*	*-17*	*4-HDHA*
				CXP	*-15*	*-15*
Q1 Mass (Da)	***Q3 Mass (Da)***	***RT (min)***	***Param***	***Start***	***Stop***	***ID***
345.2	*257.1*	*17.5*	*CE*	*-17*	*-17*	*21-HDPA*
				CXP	*-14*	*-14*
Q1 Mass (Da)	***Q3 Mass (Da)***	***RT (min)***	***Param***	***Start***	***Stop***	***ID***
345.2	*247.1*	*17.5*	*CE*	*-17*	*-17*	*17-HDPA*
				CXP	*-14*	*-14*

Q1 Mass (Da)	***Q3 Mass (Da)***	***RT (min)***	***Param***	***Start***	***Stop***	***ID***
345.2	*207.1*	*17.5*	*CE*	*-17*	*-17*	*14-HDPA*
				CXP	*-14*	*-14*
Q1 Mass (Da)	***Q3 Mass (Da)***	***RT (min)***	***Param***	***Start***	***Stop***	***ID***
345.2	*195.1*	*17.4*	*CE*	*-17*	*-17*	*13-HDPA*
				CXP	*-14*	*-14*

Source Data

Q1 Mass (Da)	***Q3 Mass (Da)***	***RT (min)***	***Param***	***Start***	***Stop***	***ID***
345.2	*143.1*	*17.5*	*CE*	*-18*	*-18*	*7-HDPA*
				CXP	*-15*	*-15*
Q1 Mass (Da)	***Q3 Mass (Da)***	***RT (min)***	***Param***	***Start***	***Stop***	***ID***
317.2	*259.1*	*15.9*	*CE*	*-16*	*-16*	*18-HEPE*
				CXP	*-23*	*-23*
Q1 Mass (Da)	***Q3 Mass (Da)***	***RT (min)***	***Param***	***Start***	***Stop***	***ID***
317.2	*219.1*	*16*	*CE*	*-18*	*-18*	*15-HEPE*
				CXP	*-12*	*-12*
Q1 Mass (Da)	***Q3 Mass (Da)***	***RT (min)***	***Param***	***Start***	***Stop***	***ID***
317.2	*179.1*	*16.5*	*CE*	*-19*	*-19*	*12-HEPE*
				CXP	*-12*	*-12*
Q1 Mass (Da)	***Q3 Mass (Da)***	***RT (min)***	***Param***	***Start***	***Stop***	***ID***
317.2	*167.1*	*16.1*	*CE*	*-19*	*-19*	*11-HEPE*
				CXP	*-12*	*-12*
Q1 Mass (Da)	***Q3 Mass (Da)***	***RT (min)***	***Param***	***Start***	***Stop***	***ID***
317.2	*115.1*	*17.3*	*CE*	*-18*	*-18*	*5-HEPE*
				CXP	*-12*	*-12*
Q1 Mass (Da)	***Q3 Mass (Da)***	***RT (min)***	***Param***	***Start***	***Stop***	***ID***
319.2	*219.1*	*17.2*	*CE*	*-19*	*-19*	*15-HETE*
				CXP	*-12*	*-12*
Q1 Mass (Da)	***Q3 Mass (Da)***	***RT (min)***	***Param***	***Start***	***Stop***	***ID***
319.2	*179.1*	*17.4*	*CE*	*-21*	*-21*	*12-HETE*
				CXP	*-12*	*-12*
Q1 Mass (Da)	***Q3 Mass (Da)***	***RT (min)***	***Param***	***Start***	***Stop***	***ID***
319.2	*167.1*	*17.3*	*CE*	*-21*	*-21*	*11-HETE*
				CXP	*-12*	*-12*
Q1 Mass (Da)	***Q3 Mass (Da)***	***RT (min)***	***Param***	***Start***	***Stop***	***ID***
319.2	*115.1*	*17.5*	*CE*	*-21*	*-21*	*5-HETE*
				CXP	*-12*	*-12*

Source Data

Q1 Mass (Da)	*Q3 Mass (Da)*	*RT (min)*	*Param*	*Start*	*Stop*	*ID*
303.3	*259.1*	*18.5*	*CE*	*-16*	*-16*	*AA*
				CXP	*-18*	*-18*
Q1 Mass (Da)	***Q3 Mass (Da)***	***RT (min)***	***Param***	***Start***	***Stop***	***ID***
301.3	*257.1*	*17.9*	*CE*	*-16*	*-16*	*EPA*
				CXP	*-18*	*-18*
Q1 Mass (Da)	***Q3 Mass (Da)***	***RT (min)***	***Param***	***Start***	***Stop***	***ID***
327.3	*283.1*	*18.5*	*CE*	*-16*	*-16*	*DHA*
				CXP	*-18*	*-18*
Q1 Mass (Da)	***Q3 Mass (Da)***	***RT (min)***	***Param***	***Start***	***Stop***	***ID***
329.3	*285.1*	*19*	*CE*	*-16*	*-16*	*DPA*
				CXP	*-18*	*-18*
Q1 Mass (Da)	***Q3 Mass (Da)***	***RT (min)***	***Param***	***Start***	***Stop***	***ID***
306	*221*	*0.6*	*CE*	*-16*	*-16*	*ignore*
				CXP	*-18*	*-18*
Q1 Mass (Da)	***Q3 Mass (Da)***	***RT (min)***	***Param***	***Start***	***Stop***	***ID***
323.2	*235.1*	*11.3*	*CE*	*-20*	*-20*	*Beta Ox LXA4*
				CXP	*-13*	*-13*
Q1 Mass (Da)	***Q3 Mass (Da)***	***RT (min)***	***Param***	***Start***	***Stop***	***ID***
325.2	*237.1*	*11.3*	*CE*	*-20*	*-20*	*d2 Beta Ox LXA4*
				CXP	*-13*	*-13*

22. Deuterium-labeled lipid mediators mark the reverse phase LC chromatographic behaviors for d_4-PGE_2 used for PGD_2, PGE_2, $PGF_{2α}$, and TXB_2; d_5-LXA_4 used for trihydroxy-containing LXA_4, AT-LXA_4, LXB_4, AT-LXB_4, LXA_5, LXB_5, RvE1; d_4-LTB_4 used for dihydroxy-containing 5S,15S-diHETE, 5R,6S-diHETE, RvD5, RvD6, RvE2, RvE3, PD1, AT-PD1, PDX, LTB_4, 20-OH-LTB_4, 20-COOH-LTB_4, 22-OHPD1, MaR1, 7S,14S-diHDHA, 4,14-HDHA, 5S,12S-diHETE; d_8-5S-HETE used for monohydroxy-containing 17-HDHA, 14-HDHA, 7-HDHA, 4-

HDHA, 15-HETE, 12-HETE, 5-HETE, 18-HEPE, 15-HEPE, 12-HEPE, 5-HEPE, AA, EPA, DHA; d_5RvD_2 used for trihydroxy-containing RvD1, AT-RvD1, RvD2, RvD3, AT-RvD3 and RvD4.

23. Each lipid mediator is identified according to published physical criteria that include at least six diagnostic ions (from EPI scans) consistent with the structure of the lipid mediator.

24. Amounts of each mediator are calculated using the following formula:

$$X\ (pg) = \frac{a}{b} \times c\ (pg)$$

where;

X = lipid mediator quantity after extraction recovery calculation (pg)

a = area beneath the peak of deuterium-label in system calibration internal standard 100%

b = area beneath the peak of deuterium-label in the sample

c = lipid mediator quantity in sample before extraction recovery calculation (pg)

• **Step-by-step data processing and graph creation (for Figure 1a)**

(A) Top panel: Multiple Reaction Monitoring (MRM) **chromatograms** of RvD2 and RvD5 from standard mixture and from human macrophages incubated with *E. coli*

1. Data were acquired and analyzed using Analyst software Version 1.6.2 (Framingham, Massachusetts) for LC-MS-MS (QTRAP 5500 AB Sciex equipped with LC-20AD HPLC Shimadzu).

2. Chromatographic behaviors are matched with those of synthetic standards for each lipid mediator. In this case in Figure 1a, RvD2 elutes at 11.0 min and RvD5 elutes at 13.7 min. See “Step-by-step solid phase extraction and LCMS-MS of lipid mediators” step #20 screen shot, green line denotes the gradient.

3. From the total multiple reaction monitoring (MRM) chromatograms, an extracted MRM transition was obtained for each mediator (see below) *RvD2 and RvD5 in the standard mixture (direct screen capture from Analyst software)*

Source Data

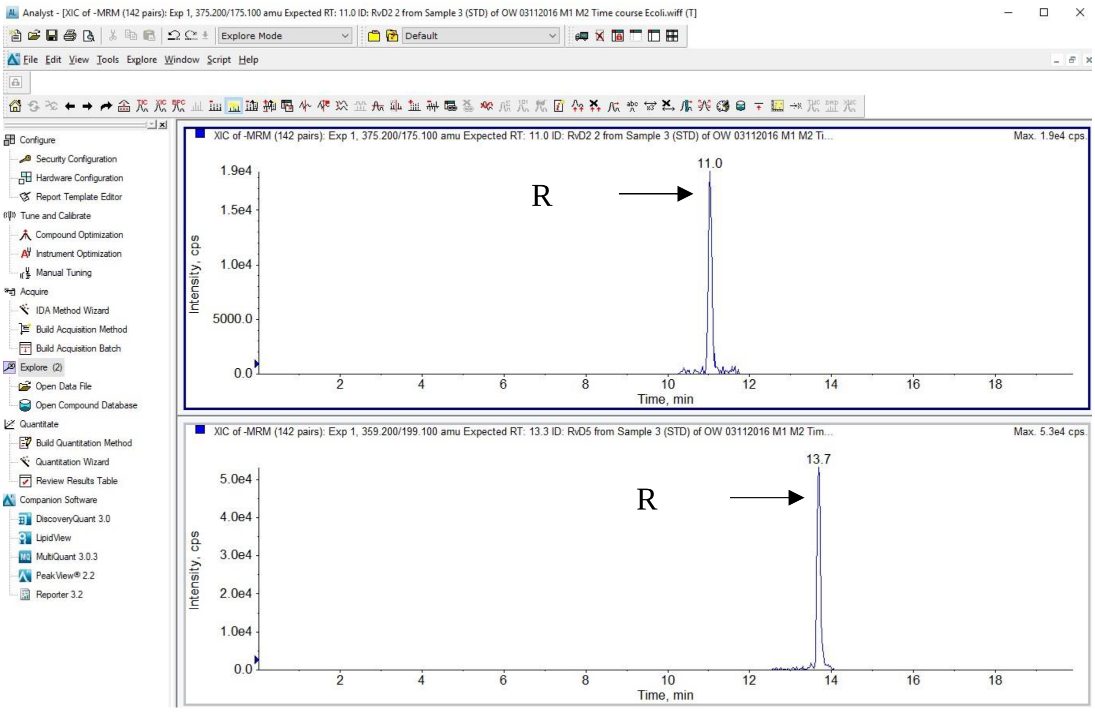

RvD2 and RvD5 obtained from human macrophages incubated with E. coli (direct screen capture from Analyst software)

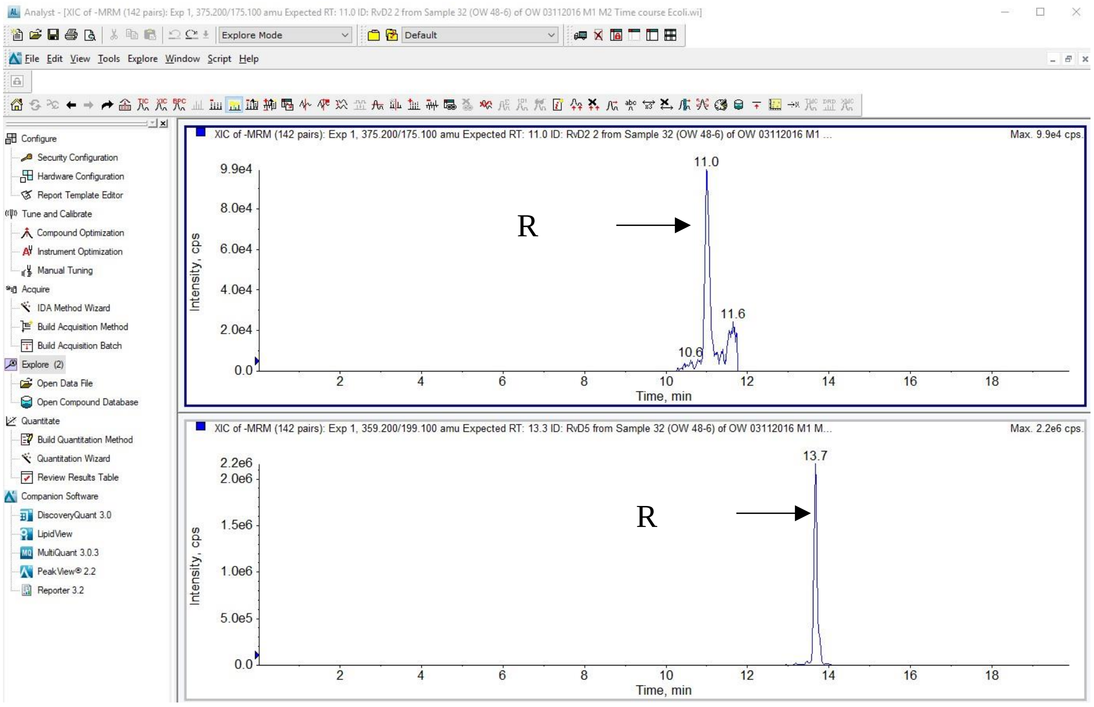

4. Retention time (RT) from 10.0-12.0 mins for RvD2 and 13.2-14.5 min for RvD5 were enlarged and presented in insets in Figure 1a top panels (screen captures)

Source Data

RvD2 in the standard mixture RvD2 in MΦ incubation with *E.coli*

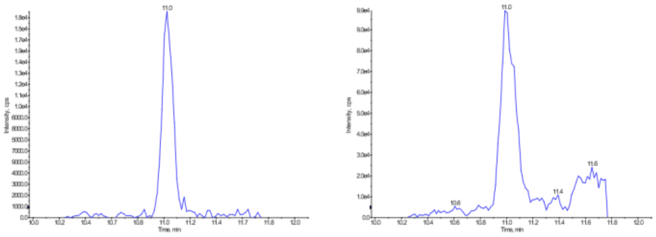

RvD5 in the standard mixture RvD5 in MΦ incubation with *E.coli*

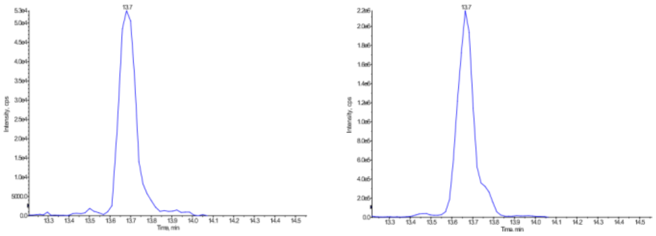

5. Final figure 1a (top panels)

a

Multiple Reaction Monitoring (MRM) chromatograms of RvD2 and RvD5

RvD2 in the standard mixture RvD2 in MΦ incubation with *E.coli*

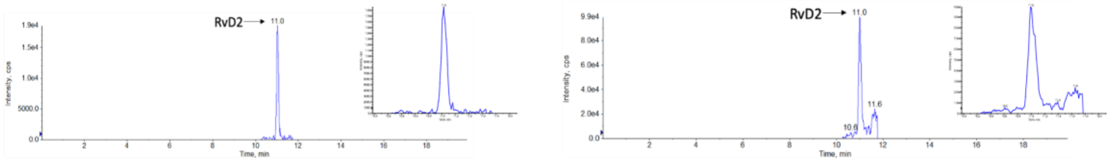

RvD5 in the standard mixture RvD5 in MΦ incubation with *E.coli*

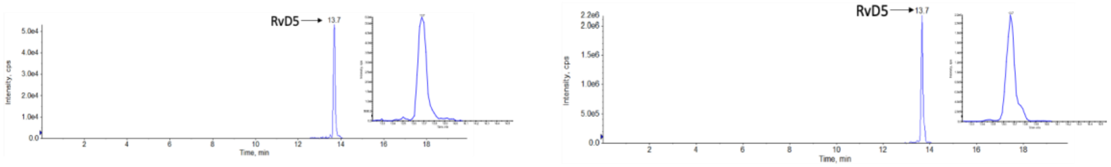

(B) Bottom panels: **MS-MS spectra** of RvD2 and RvD5 obtained from human macrophages incubated with *E. coli*

1. We obtained MS-MS Spectra from LC-MS-MS software Analyst Version
1. 6.2; Framingham, MA. The scan interval was between ratio mass/charge 100 to 450 *m/z,* Da. Each mediator was identified using published criteria obtained in their structure elucidation, which included identification criteria of at least six characteristic diagnostic ions present in their MS-MS spectra.

2. From the total ion chromatogram (TIC), the enhanced product ion (EPI) spectra were extracted at the RT=11.0 min for RvD2 and RT=13.7 for RvD5

3. Centroided spectra from Analyst software 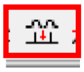

We then used Analyst software Version 1.6.2 that performs an automatic baseline subtraction in order to remove a constant or slowly varying offset from a set of data, facilitating the location of small peaks that are obscured by noise. The noise threshold is the level below which data is considered noise. In a graph, the noise threshold is a line parallel to the x-axis denoted by a blue arrowhead to the left of the y-axis representing the noise threshold.

We also applied the “centroid” feature in the Analyst software Version 1.6.2. Centroiding a spectrum converts peak distribution values into a single value that represents the peak. The algorithm used by the software converts peaks to single values by using an intensity-weighted average to calculate the center of gravity of the peak.

RvD2 – EPI spectrum centroided (direct screen capture from Analyst software)

Source Data

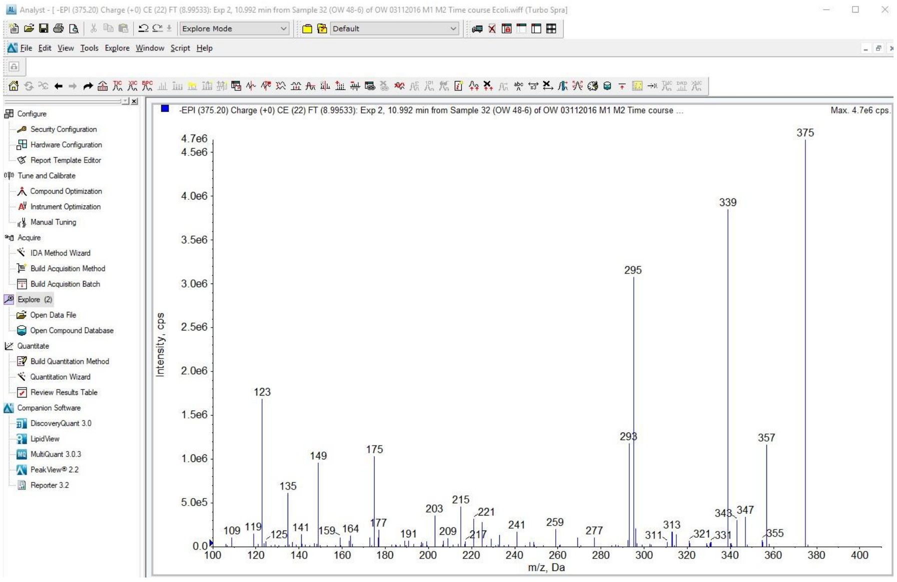

RvD5 – EPI spectrum centroided (direct screen capture from Analyst software)

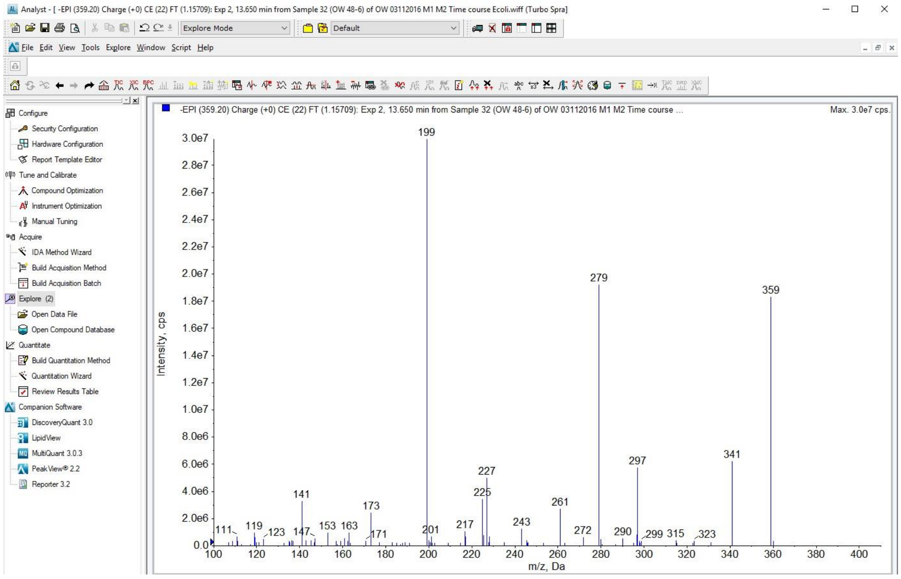

4. RvD2 and RvD5 were identified by matching at least six characteristic diagnostic ions present in the MS-MS spectra.

5. We then listed diagnostic ions and presented chemical structures of RvD2 and RvD5 with proposed fragmentations, which are shown in the schematic illustrations in the insets.

Source Data

MS-MS spectra

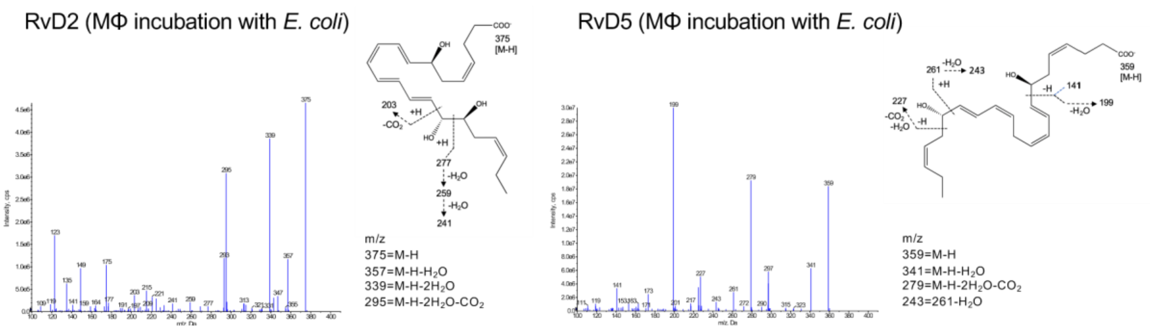

The ions used for identification of RvD2 included m/z 375, 357, 339, 295, 277, 259, 241, 203.

The ions used for identification of RvD5 included m/z 359, 341, 279, 261, 243, 227, 199, 141.

Following in the spirit of this author correction, we are providing the original scans of 3 Western blot images presented in Fig. 3b and Supplementary Fig. 3. Please note that all Western blots presented in Fig. 3b and Supplementary Fig. 3 are original scans of single membranes without cutting and are single blots. These were carried out at the University of Jena, Germany.

Source Data

Source data: Chromatographs and mass spectra of RvD2 and RvD5

Donor 1

File name: OW 03112016 M1 M2 Time course Ecoli.wiff Sample (STD) and (OW 48-6)

Donor 1: RvD2 and RvD5 standard chromatograms: OW 03112016 M1 M2 Time course Ecoli.wiff (STD)

Source Data

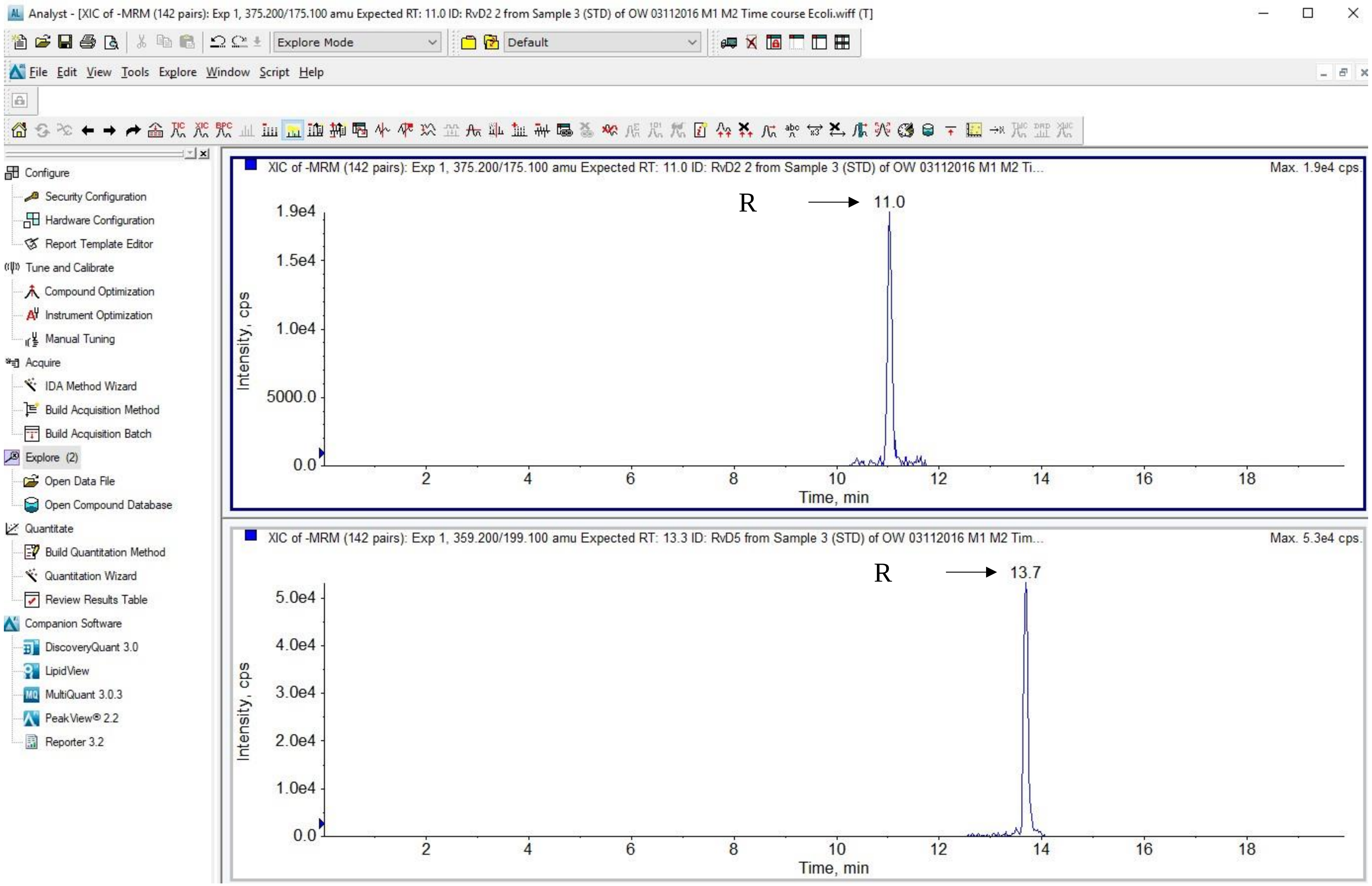

Donor 1: RvD2 and RvD5 chromatograms: OW 03112016 M1 M2 Time course Ecoli.wiff (OW 48-6)

Source Data

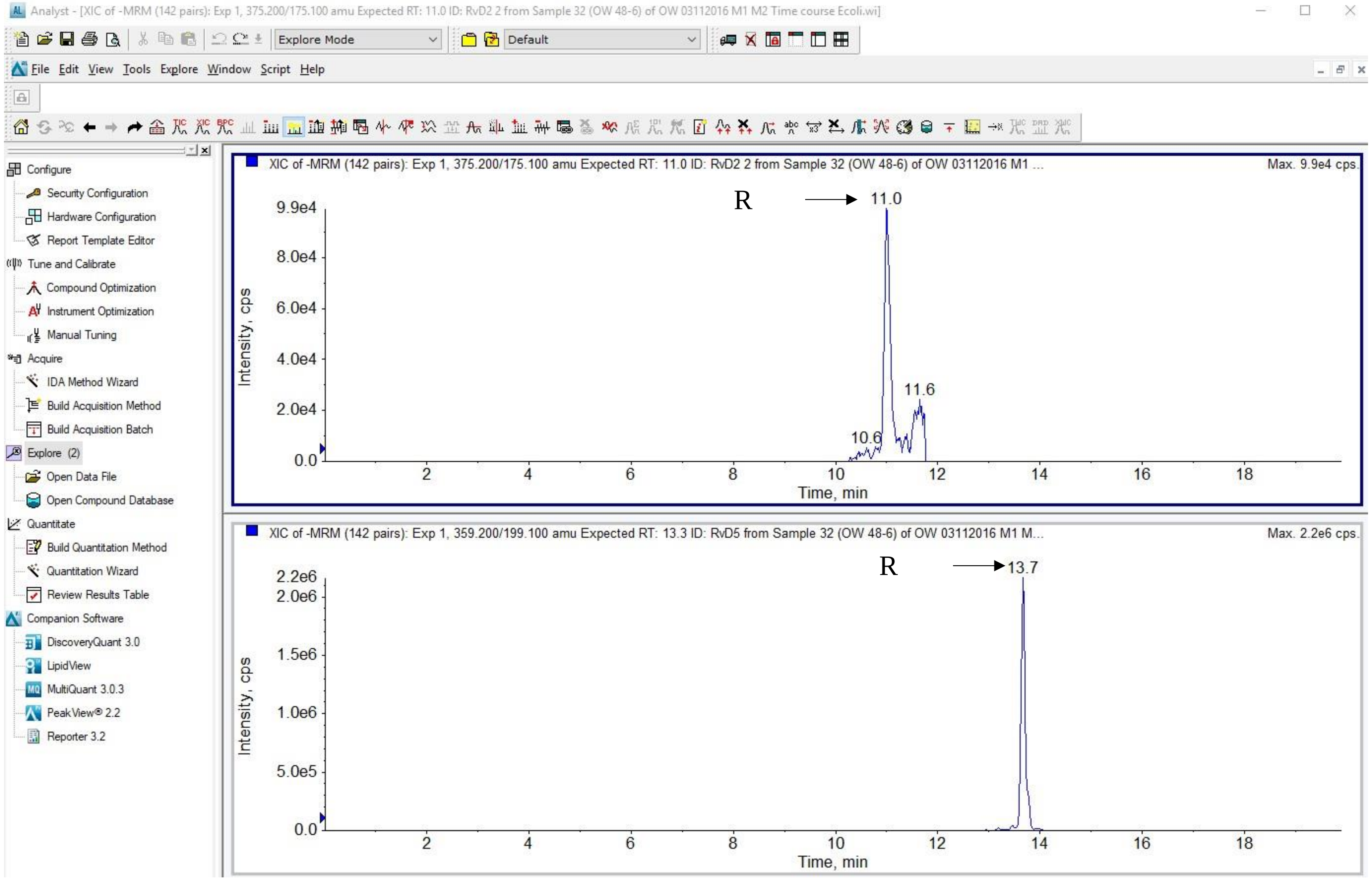

Donor 1: RvD2 EPI spectrum: OW 03112016 M1 M2 Time course Ecoli.wiff (OW 48-6)

Source Data

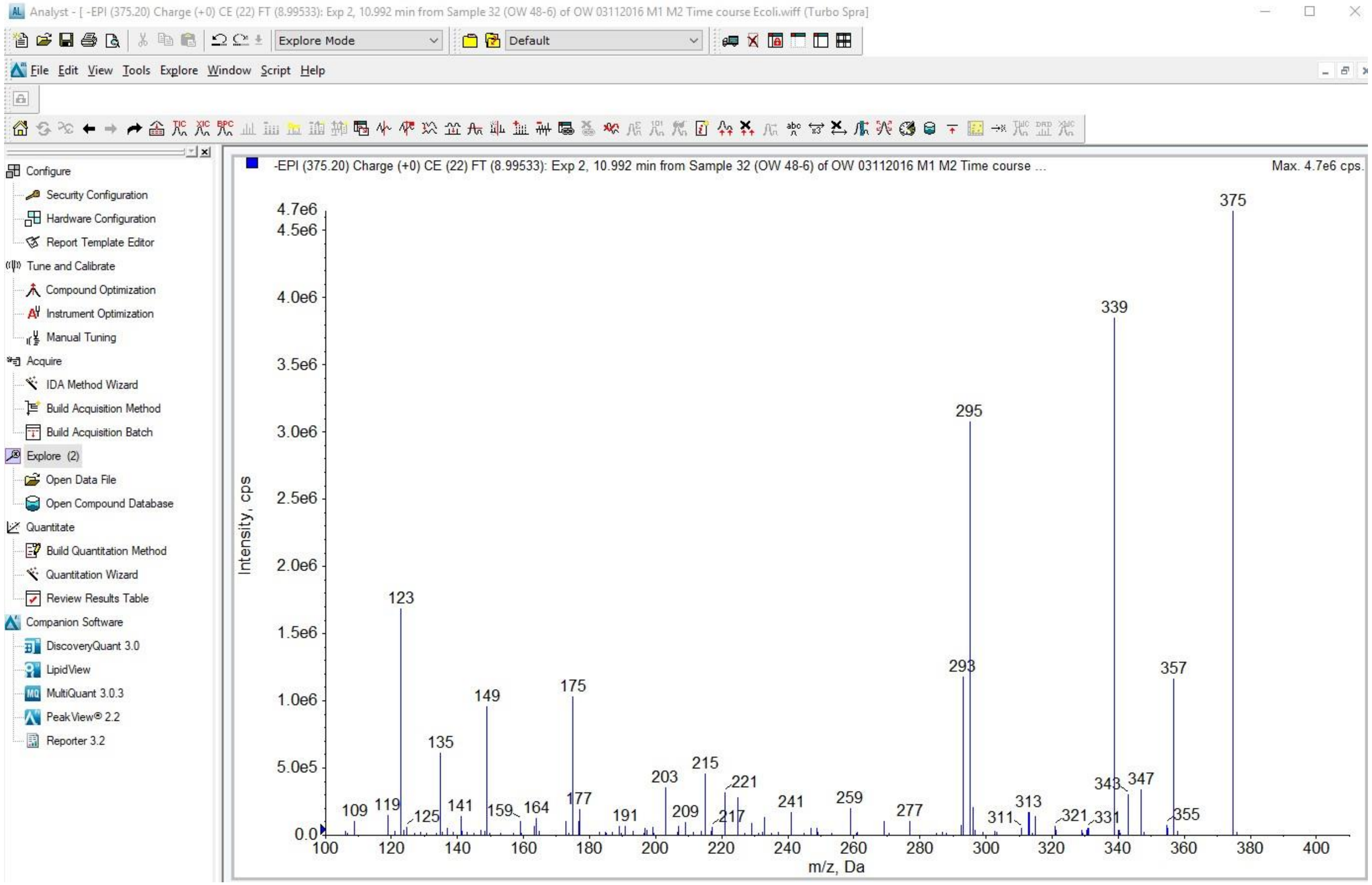

Donor 1: RvD5 EPI spectrum: OW 03112016 M1 M2 Time course Ecoli.wiff (OW 48-6)

Source Data

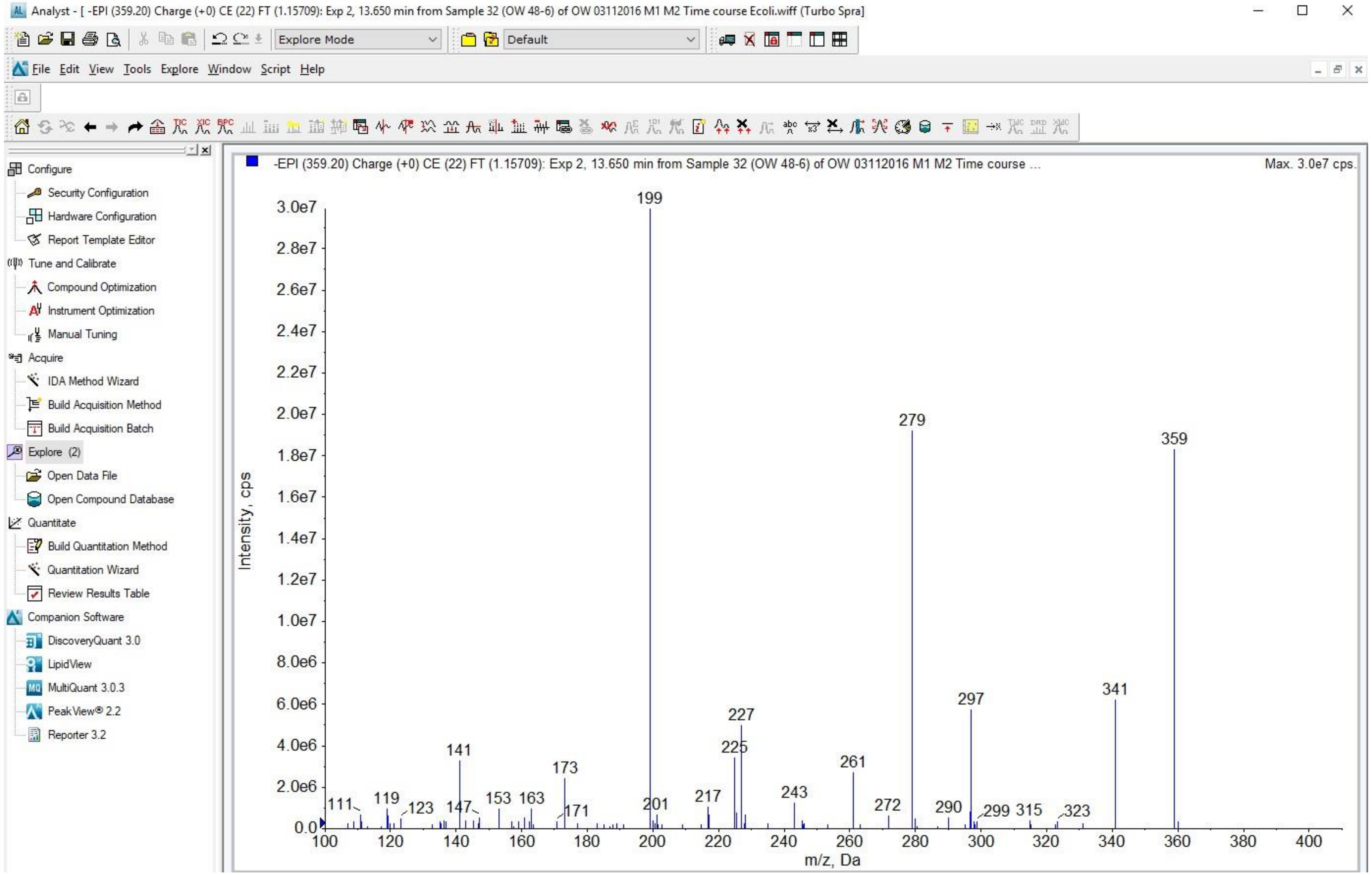
Analyst - [-EPI (359.20) Charge (+0) CE (22) FT (1.15709): Exp 2, 13.650 min from Sample 32 (OW 48-6) of OW 03112016 M1 M2 Time course Ecoli.wiff (Turbo Spra]
Explore Mode
Default
File Edit View Tools Explore Window Script Help
Configure
Security Configuration
Hardware Configuration
Report Template Editor
Tune and Calibrate
Compound Optimization
Instrument Optimization
Manual Tuning
Acquire
IDA Method Wizard
Build Acquisition Method
Build Acquisition Batch
Explore (2)
Open Data File
Open Compound Database
Quantitate
Build Quantitation Method
Quantitation Wizard
Review Results Table
Companion Software
DiscoveryQuant 3.0
LipidView
MultiQuant 3.0.3
PeakView® 2.2
Reporter 3.2
-EPI (359.20) Charge (+0) CE (22) FT (1.15709): Exp 2, 13.650 min from Sample 32 (OW 48-6) of OW 03112016 M1 M2 Time course ...
Max. 3.0e7 cps.
Intensity, cps
m/z, Da
199
279
359
341
297
227
225
261
141
173
243
217
272
290
299
315
323
111
119
123
147
153
163
171
201
3.0e7
2.8e7
2.6e7
2.4e7
2.2e7
2.0e7
1.8e7
1.6e7
1.4e7
1.2e7
1.0e7
8.0e6
6.0e6
4.0e6
2.0e6
0.0
100
120
140
160
180
200
220
240
260
280
300
320
340
360
380
400

Source Data

Donors 2 and 3

Filename: OW 03262016 M1 M2 Time course polarization.wiff Sample (STD), (OW 1-25) and (OW 1-31)

Donors 2 and 3: RvD2 and RvD5 standard chromatograms: filename: OW 03262016 M1 M2 Time course polarization.wiff

Source Data

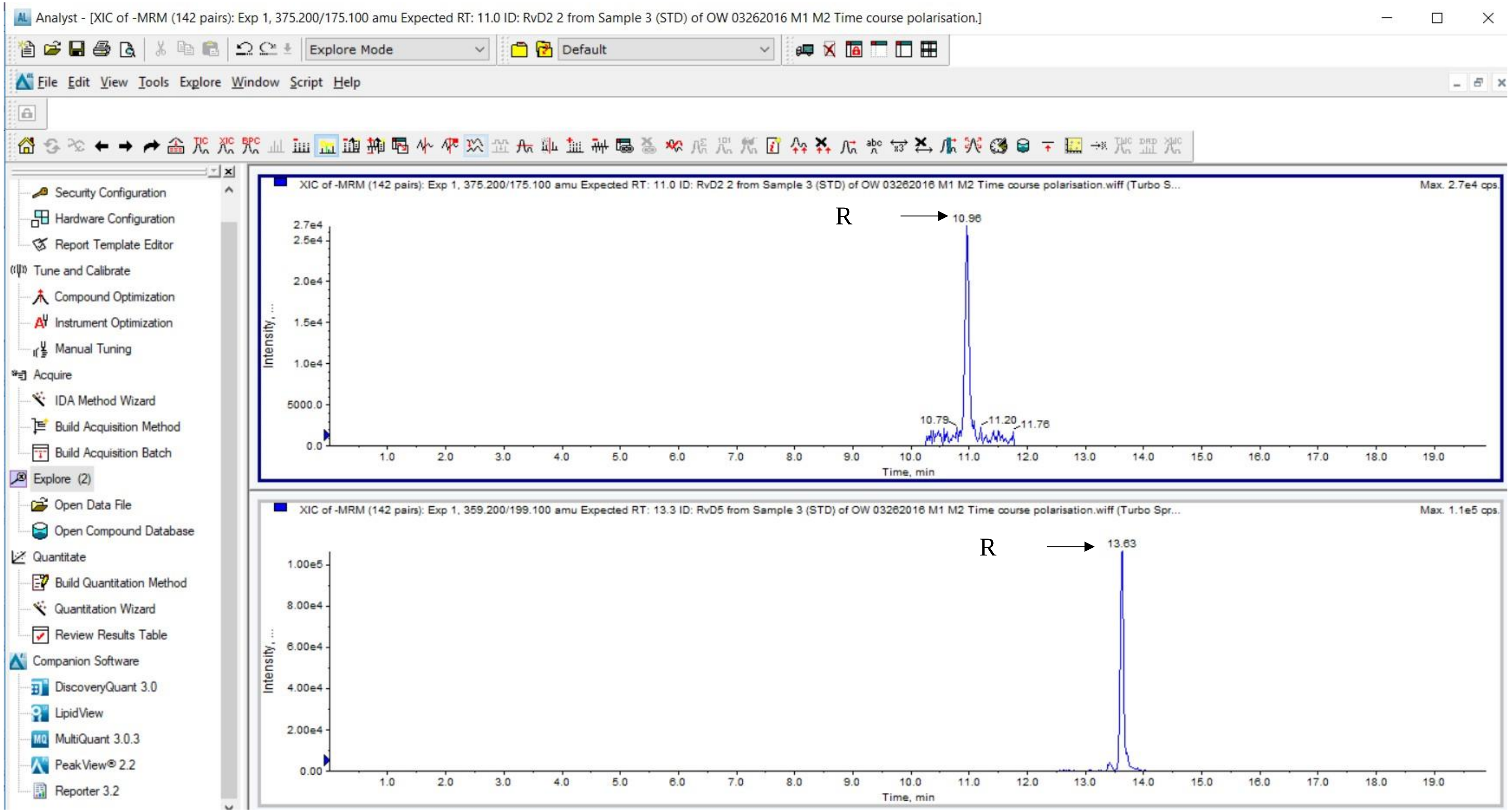

Donor 2: RvD2 and RvD5 chromatograms: filename: OW 03262016 M1 M2 Time course polarization.wiff

Source Data

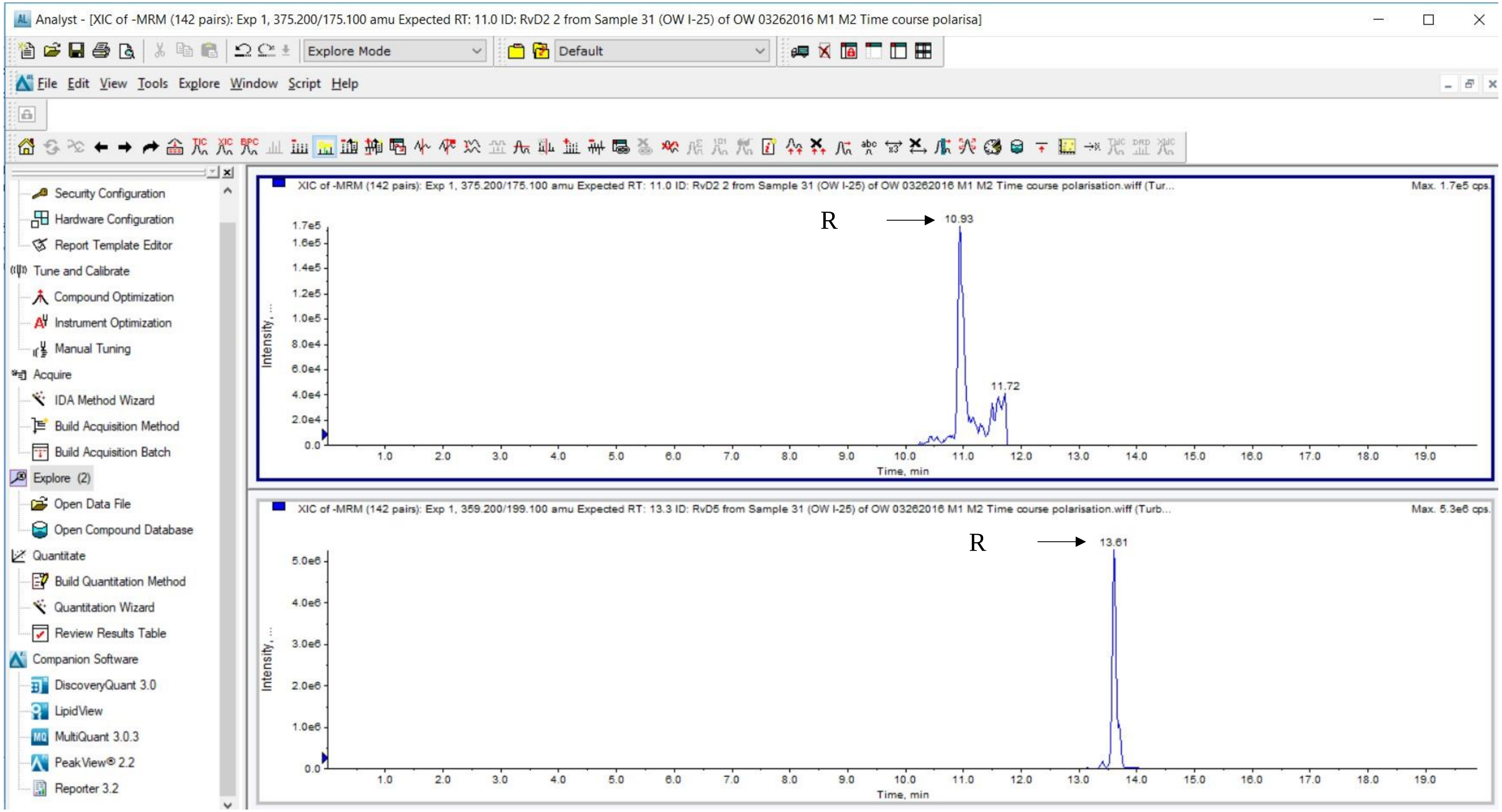

Donor 2: RvD2 EPI spectrum: OW 03262016 M1 M2 Time course polarization.wiff

Source Data

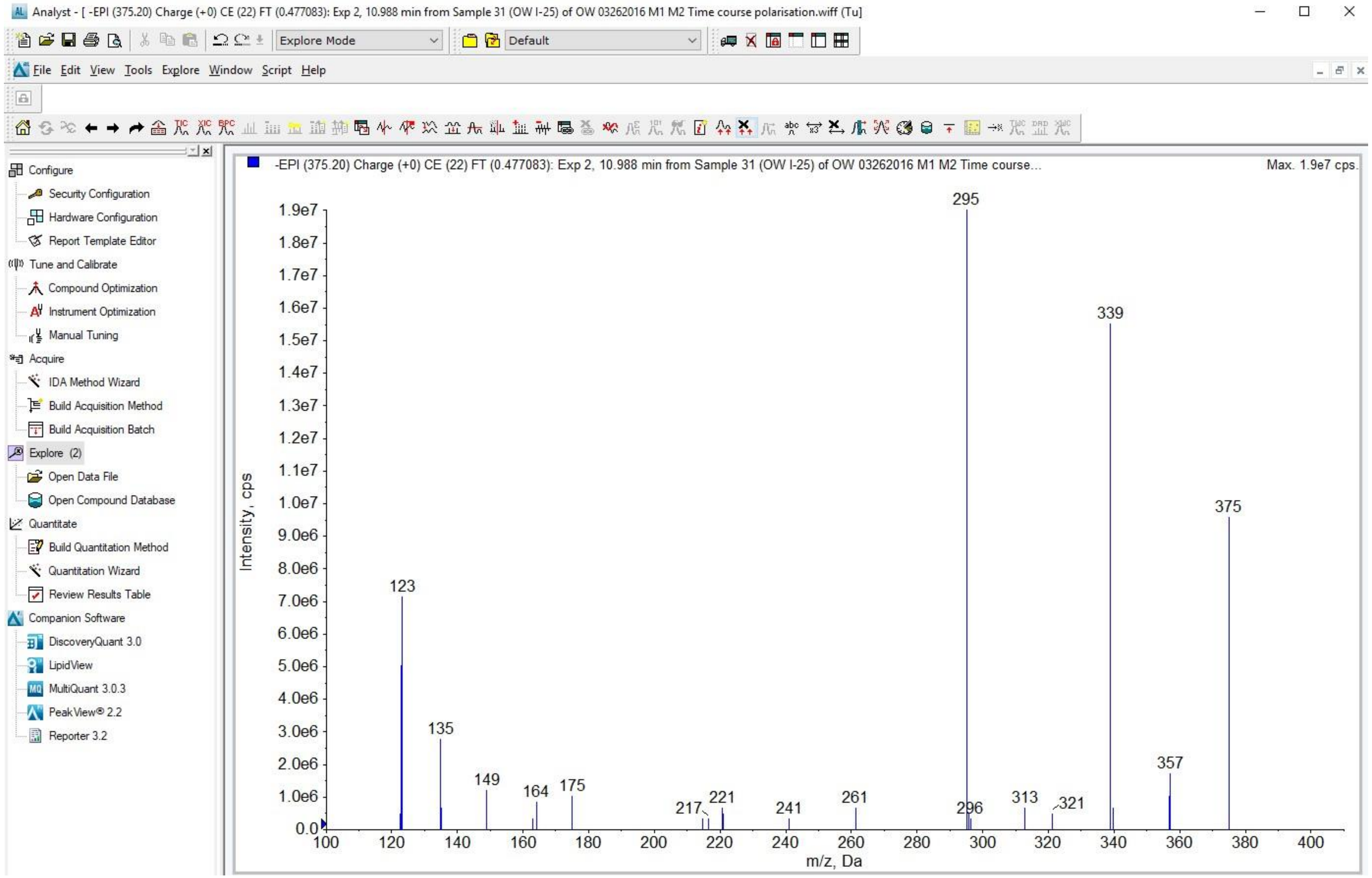

Donor 2: RvD5 EPI spectrum: OW 03262016 M1 M2 Time course polarization.wiff

Source Data

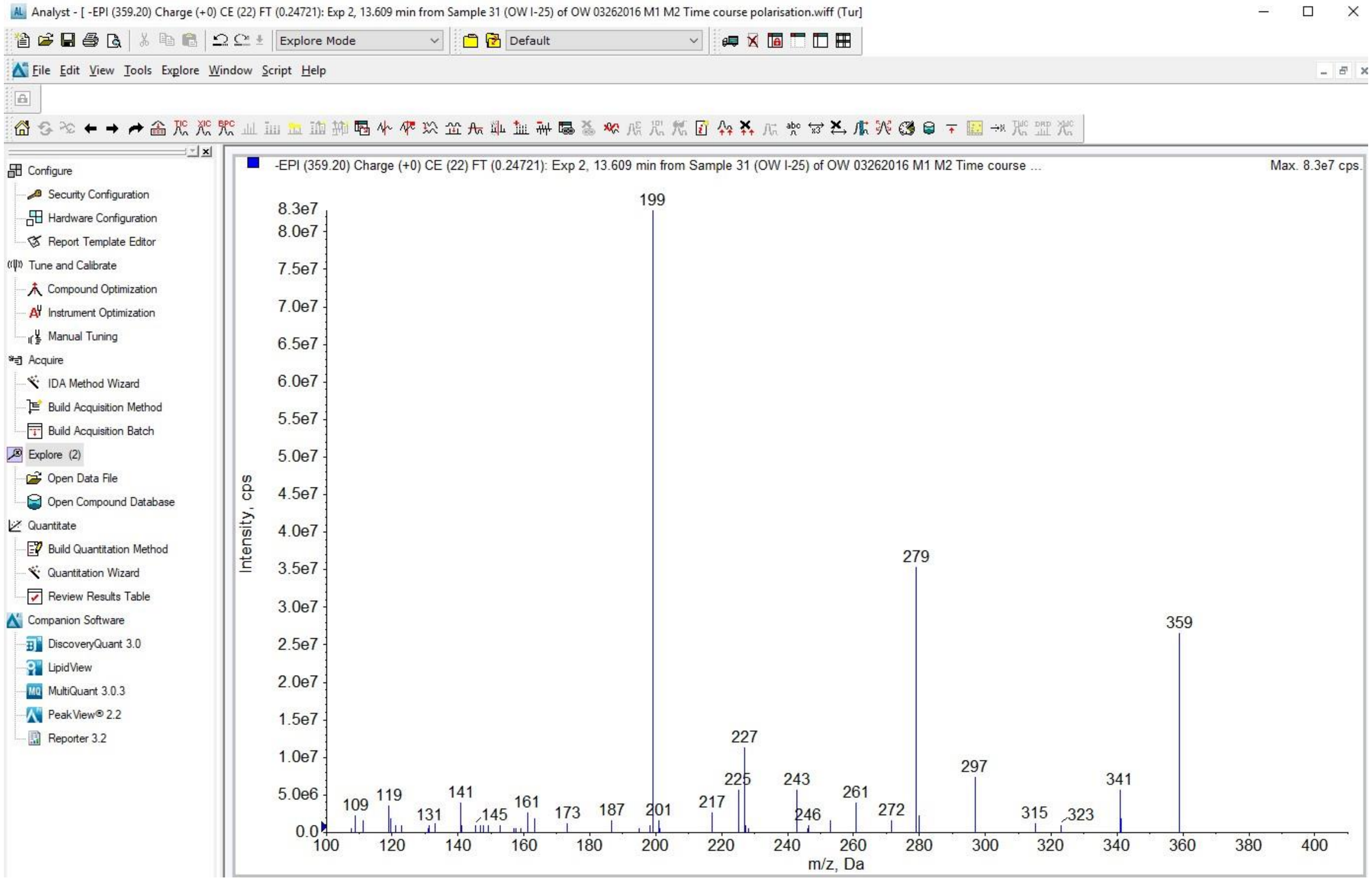

Donor 3: RvD2 and RvD5 chromatograms: OW 03262016 M1 M2 Time course polarization.wiff

Source Data

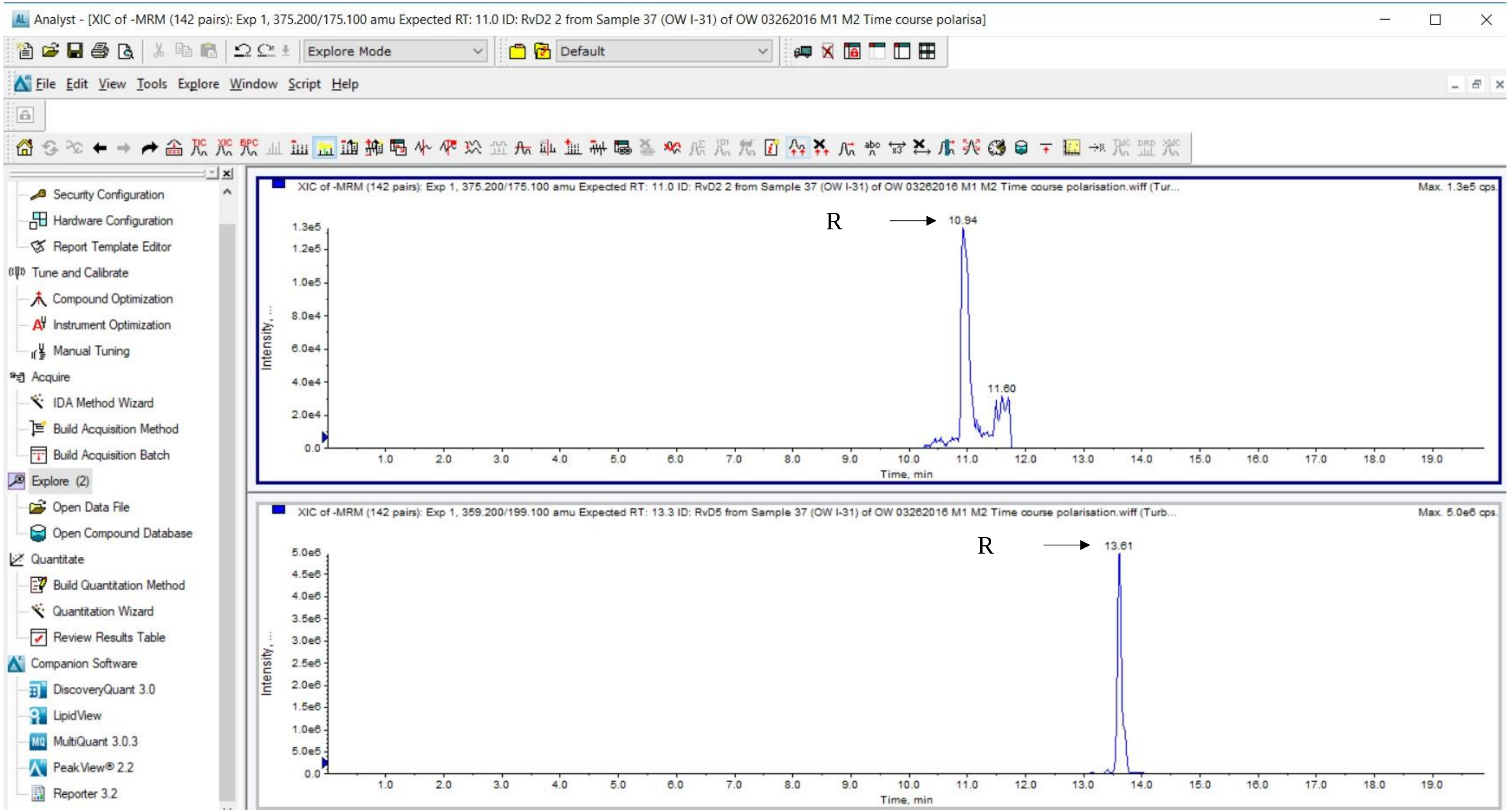

Donor 3: RvD2 EPI spectrum: OW 03262016 M1 M2 Time course polarization.wiff

Source Data

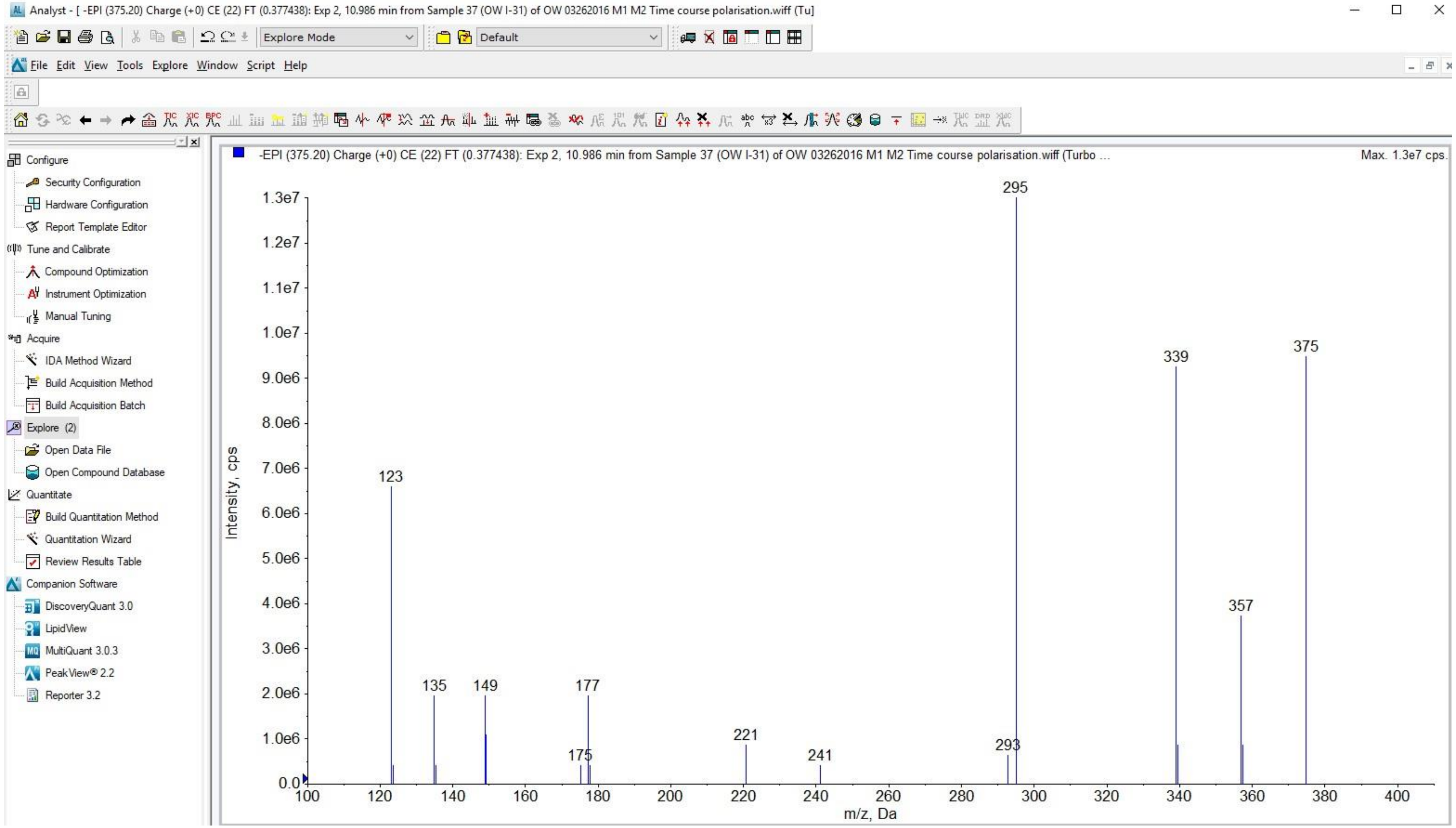

Donor 3: RvD5 EPI spectrum: OW 03262016 M1 M2 Time course polarization.wiff

Source Data

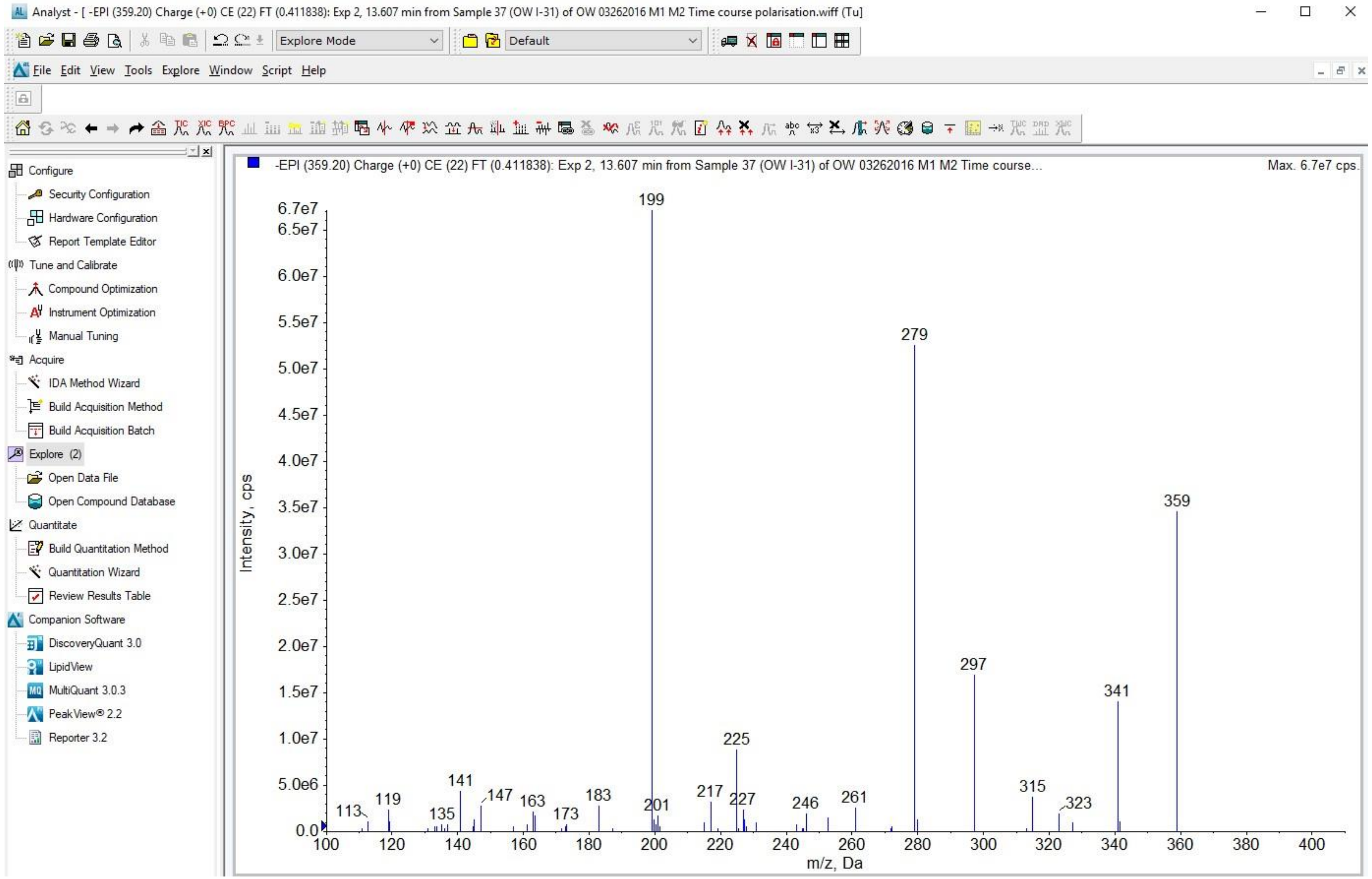

Analyst - [-EPI (359.20) Charge (+0) CE (22) FT (0.411838): Exp 2, 13.607 min from Sample 37 (OW I-31) of OW 03262016 M1 M2 Time course polarisation.wiff (Tu)]
Explore Mode
Default
File Edit View Tools Explore Window Script Help
Configure
Security Configuration
Hardware Configuration
Report Template Editor
Tune and Calibrate
Compound Optimization
Instrument Optimization
Manual Tuning
Acquire
IDA Method Wizard
Build Acquisition Method
Build Acquisition Batch
Explore (2)
Open Data File
Open Compound Database
Quantitate
Build Quantitation Method
Quantitation Wizard
Review Results Table
Companion Software
DiscoveryQuant 3.0
LipidView
MultiQuant 3.0.3
PeakView® 2.2
Reporter 3.2
-EPI (359.20) Charge (+0) CE (22) FT (0.411838): Exp 2, 13.607 min from Sample 37 (OW I-31) of OW 03262016 M1 M2 Time course...
Max. 6.7e7 cps.
Intensity, cps
m/z, Da
199
279
359
297
341
225
141
315
113
119
135
147
163
173
183
201
217
227
246
261
323

Source Data

Donor 4

Filename: OW 03182016 M1 M2 polarization RvD2 RvD5 LTB4.wiff Samples (STD) and (OW 1-7)

Donor 4: RvD2 and RvD5 **standard** chromatograms: OW 03182016 M1 M2 polarization RvD2 RvD5 LTB4.wiff (STD)

Source Data

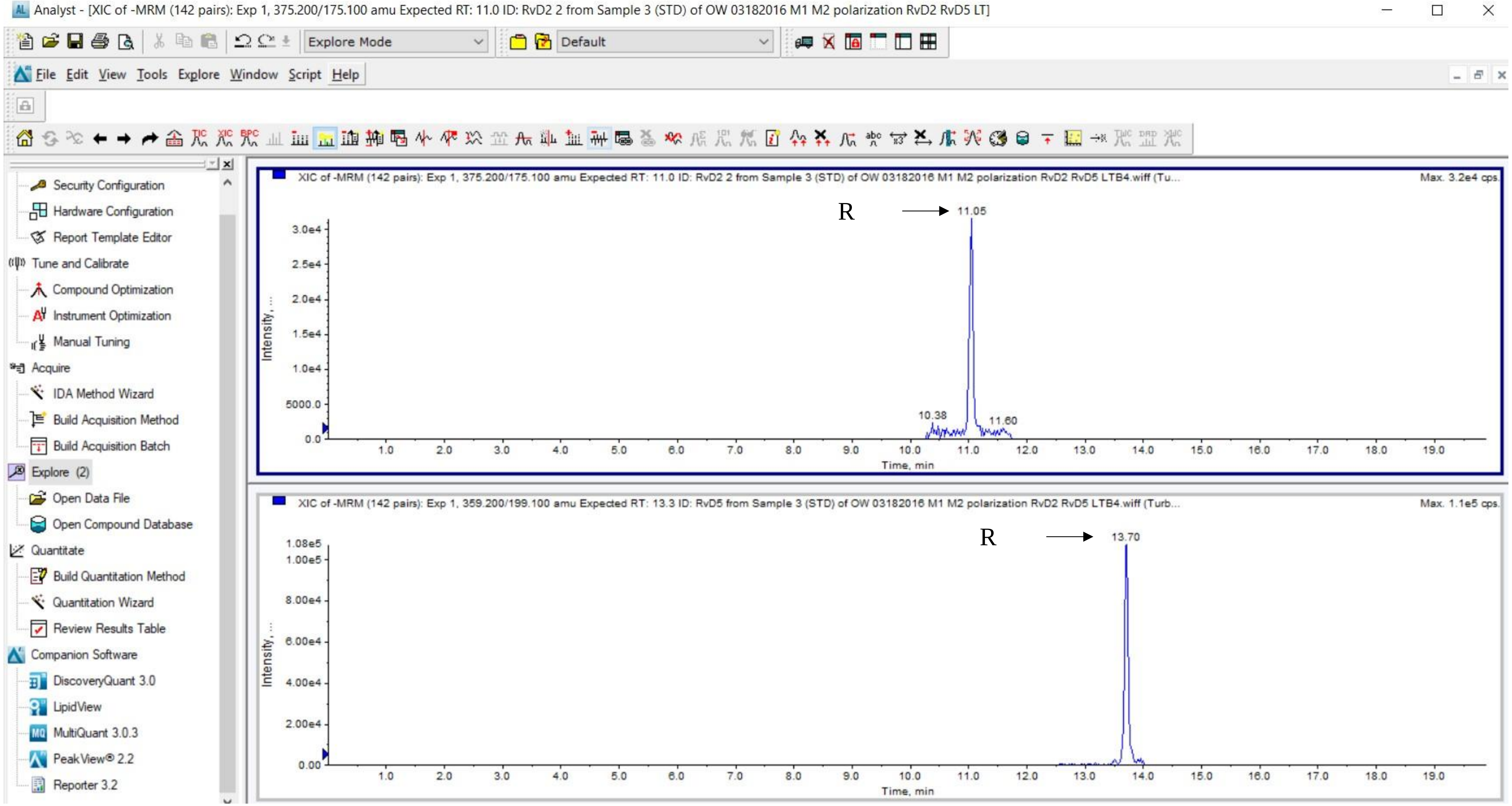

Donor 4: RvD2 and RvD5 chromatograms: OW 03182016 M1 M2 polarization RvD2 RvD5 LTB4.wiff (OW 1-7)

Source Data

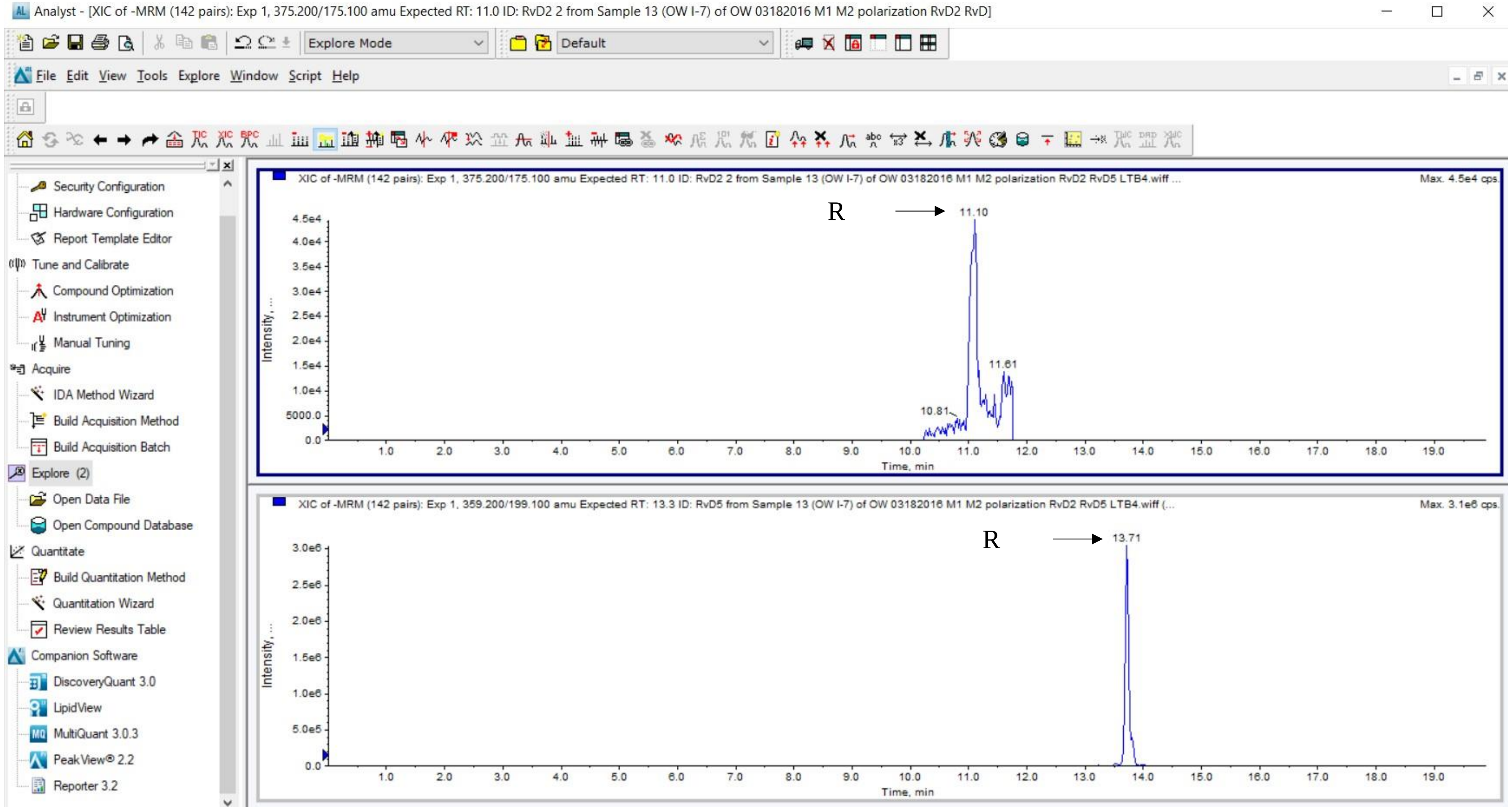

Donor 4: RvD2 EPI spectrum: OW 03182016 M1 M2 polarization RvD2 RvD5 LTB4.wiff (OW 1-7)

Source Data

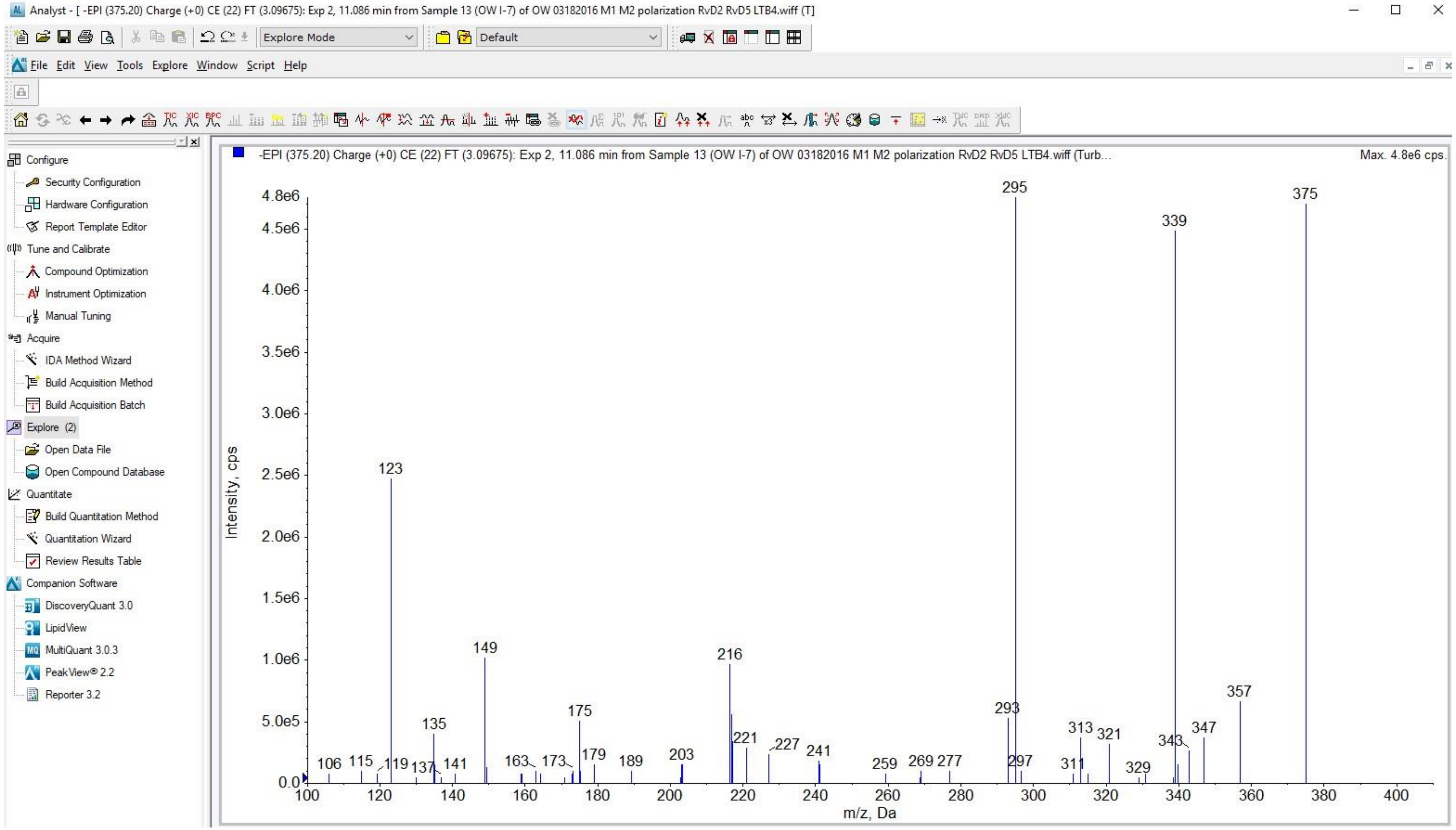

Donor 4: RvD5 EPI spectrum: OW 03182016 M1 M2 polarization RvD2 RvD5 LTB4.wiff (OW 1-7)

Source Data

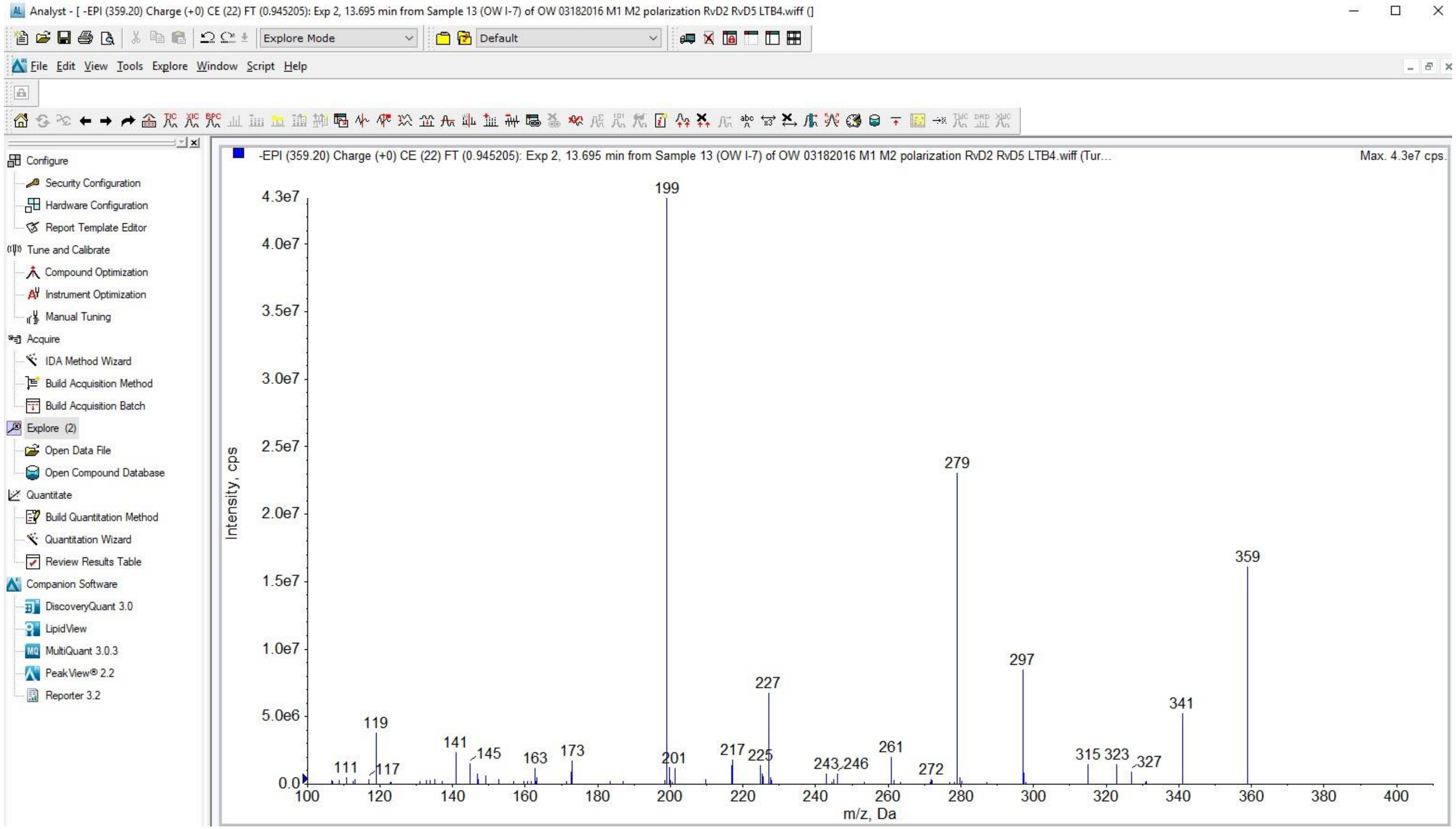

Analyst - [-EPI (359.20) Charge (+0) CE (22) FT (0.945205): Exp 2, 13.695 min from Sample 13 (OW I-7) of OW 03182016 M1 M2 polarization RvD2 RvD5 LTB4.wiff ()]
Explore Mode
Default
File Edit View Tools Explore Window Script Help
Configure
Security Configuration
Hardware Configuration
Report Template Editor
Tune and Calibrate
Compound Optimization
Instrument Optimization
Manual Tuning
Acquire
IDA Method Wizard
Build Acquisition Method
Build Acquisition Batch
Explore (2)
Open Data File
Open Compound Database
Quantitate
Build Quantitation Method
Quantitation Wizard
Review Results Table
Companion Software
DiscoveryQuant 3.0
LipidView
MultiQuant 3.0.3
PeakView® 2.2
Reporter 3.2
-EPI (359.20) Charge (+0) CE (22) FT (0.945205): Exp 2, 13.695 min from Sample 13 (OW I-7) of OW 03182016 M1 M2 polarization RvD2 RvD5 LTB4.wiff (Tur...
Max. 4.3e7 cps.
Intensity, cps
m/z, Da
199
279
359
297
227
341
119
141
145
163
173
201
217
225
261
243
246
272
315
323
327
111
117

Donor 5

File name: OW 01212016 M2 bacteria alone time course.wiff

Samples (STD), OW 1-6

Source Data

Donor 5: RvD2 and RvD5 standard chromatograms: OW 01212016 M2 bacteria alone time course.wiff (STD)

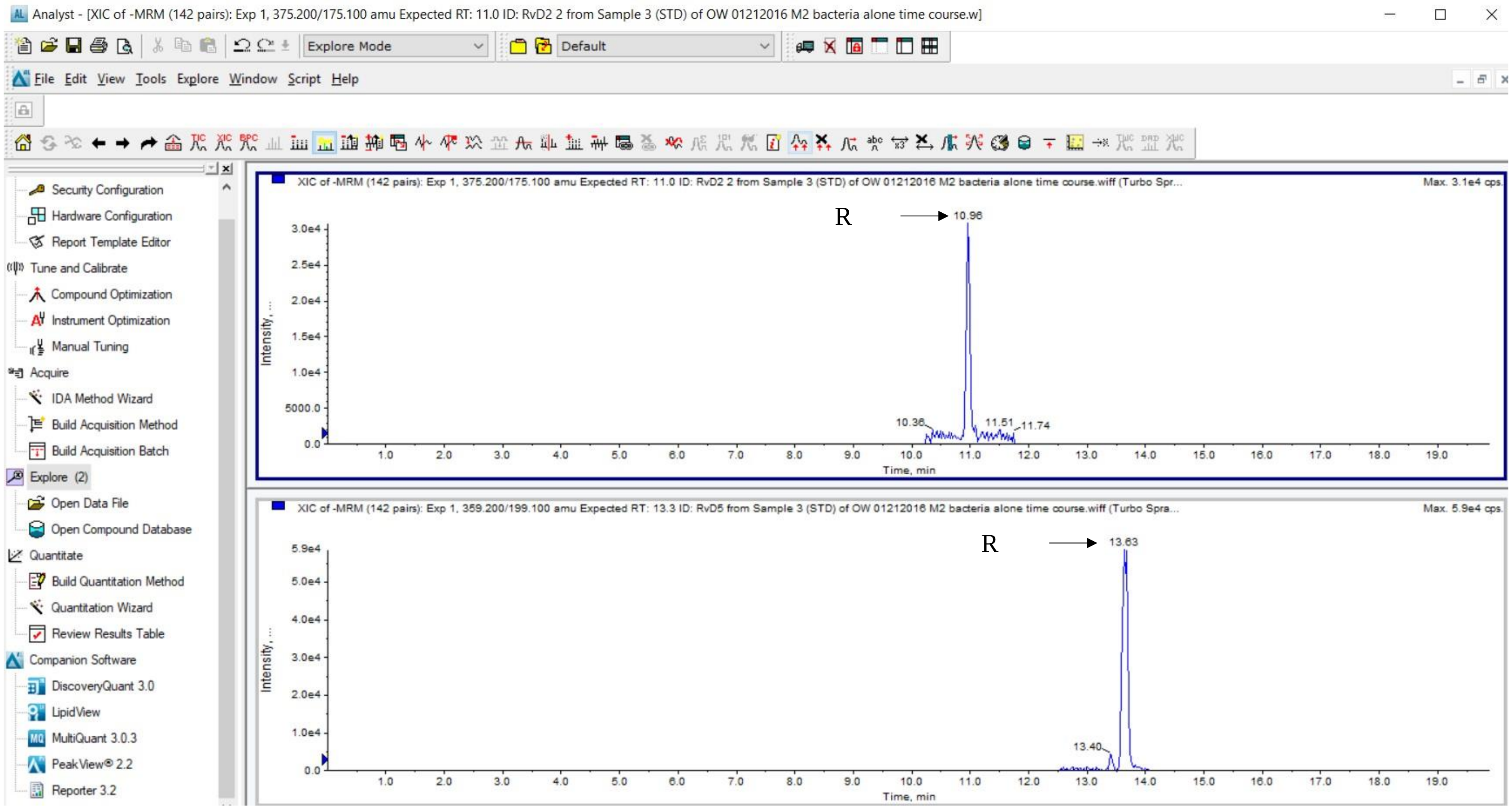

Donor 5: RvD2 and RvD5 chromatograms: OW 01212016 M2 bacteria alone time course.wiff (OW 1-6)

Source Data

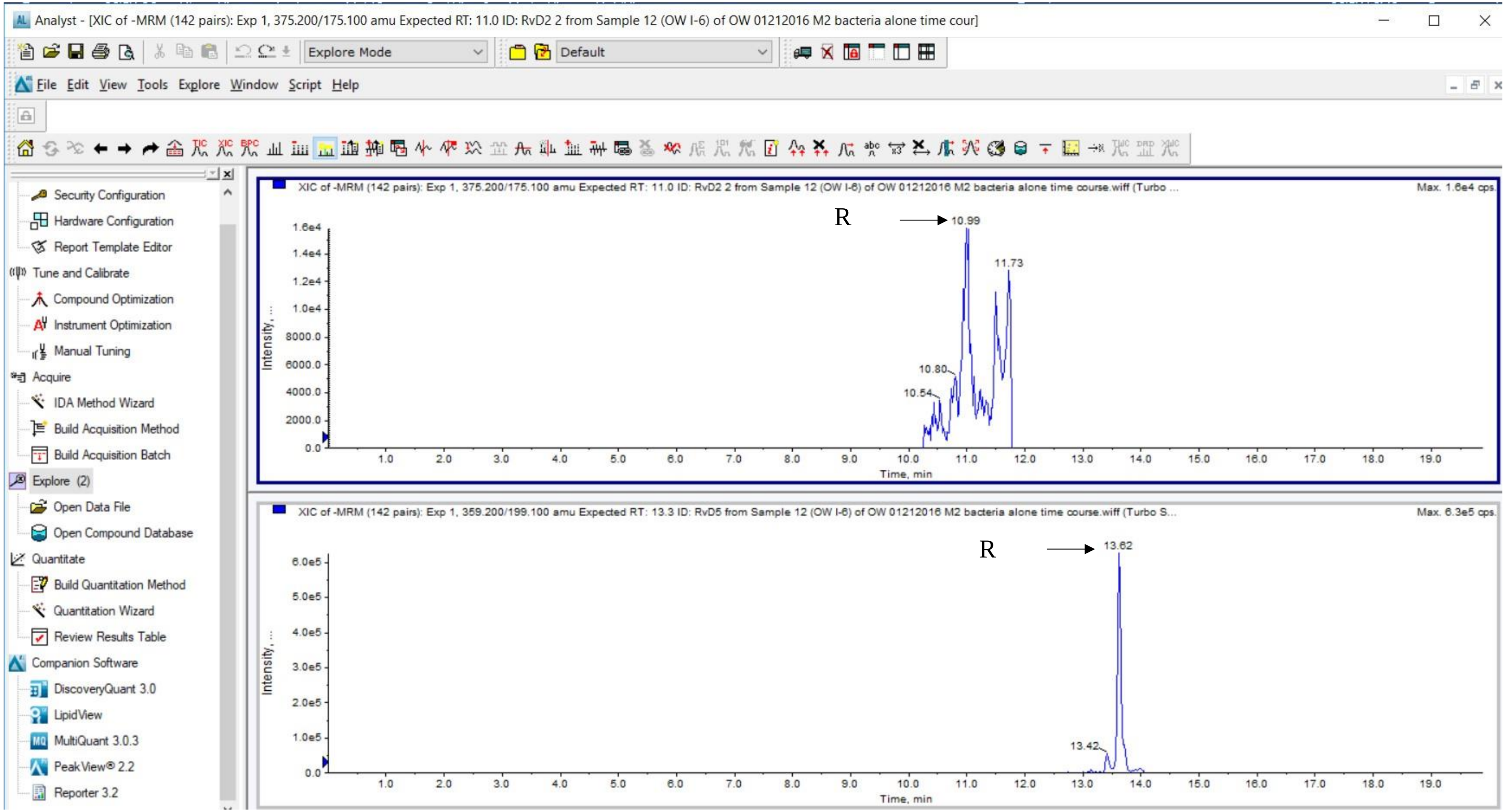

Donor 5: RvD2 EPI spectrum: OW 01212016 M2 bacteria alone time course.wiff (OW 1-6)

Source Data

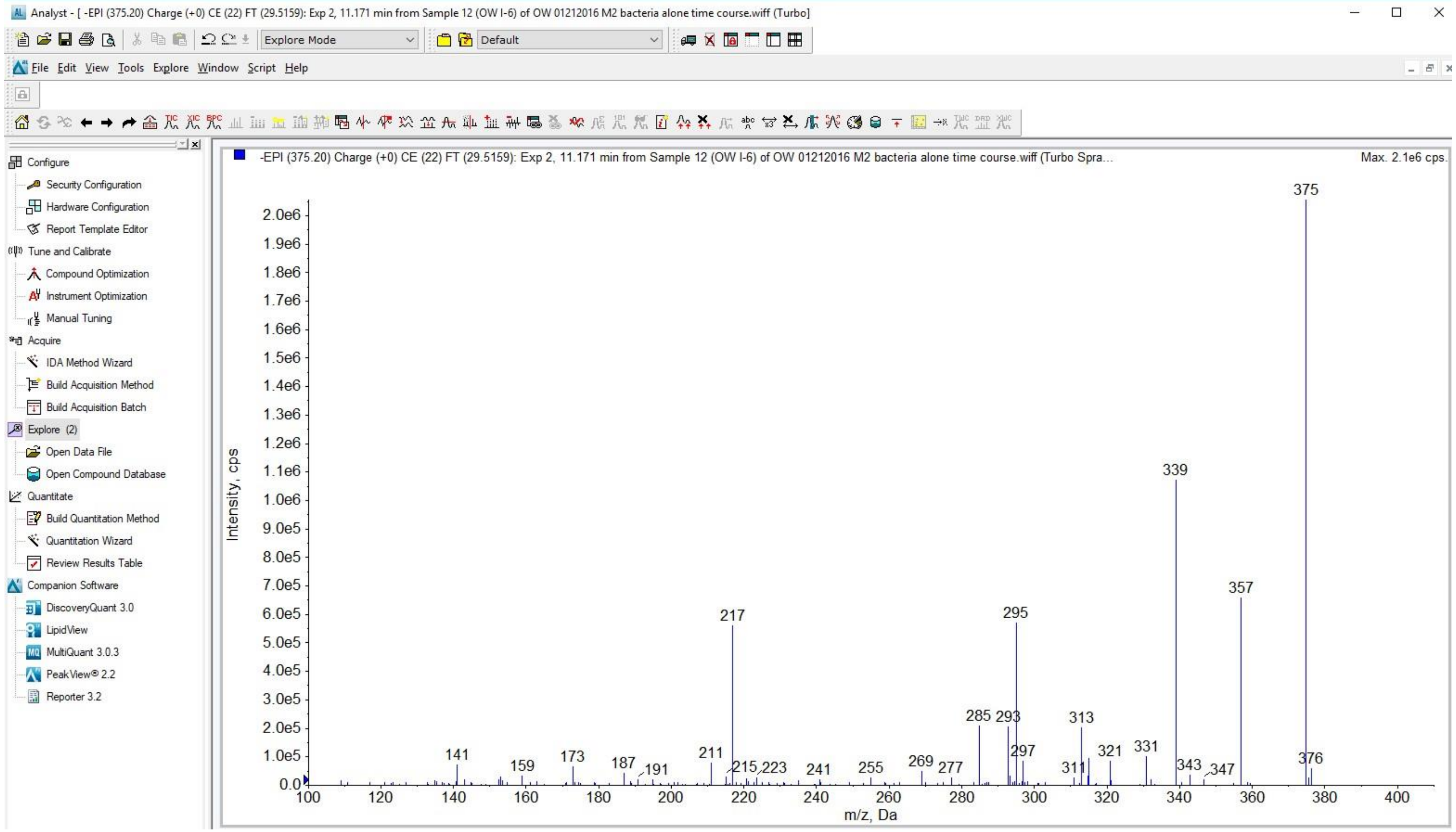

Donor 5: RvD5 EPI spectrum: OW 01212016 M2 bacteria alone time course.wiff (OW 1-6)

Source Data

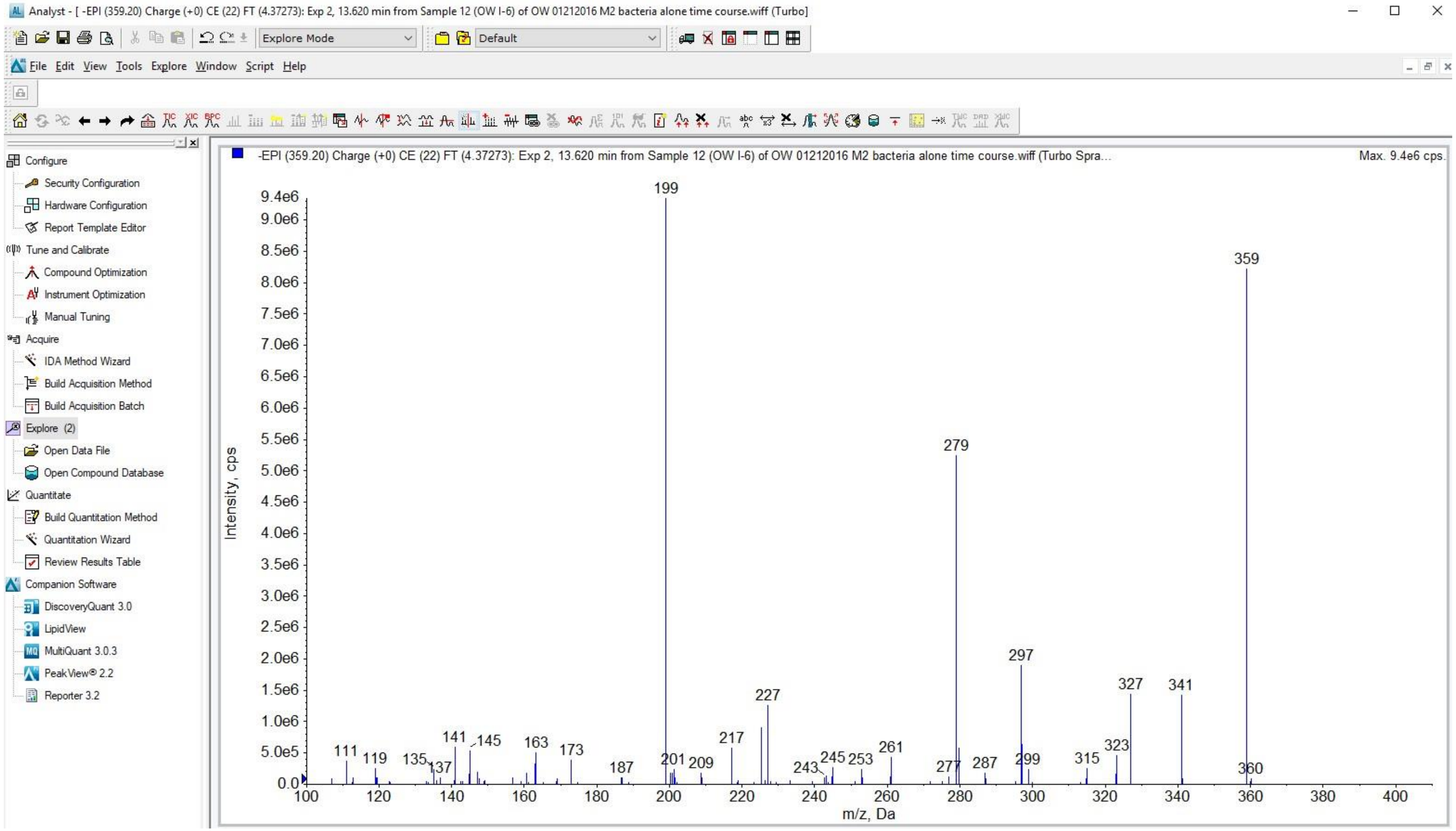
Analyst - [-EPI (359.20) Charge (+0) CE (22) FT (4.37273): Exp 2, 13.620 min from Sample 12 (OW I-6) of OW 01212016 M2 bacteria alone time course.wiff (Turbo]
Explore Mode
Default
File Edit View Tools Explore Window Script Help
Configure
Security Configuration
Hardware Configuration
Report Template Editor
Tune and Calibrate
Compound Optimization
Instrument Optimization
Manual Tuning
Acquire
IDA Method Wizard
Build Acquisition Method
Build Acquisition Batch
Explore (2)
Open Data File
Open Compound Database
Quantitate
Build Quantitation Method
Quantitation Wizard
Review Results Table
Companion Software
DiscoveryQuant 3.0
LipidView
MultiQuant 3.0.3
PeakView® 2.2
Reporter 3.2
-EPI (359.20) Charge (+0) CE (22) FT (4.37273): Exp 2, 13.620 min from Sample 12 (OW I-6) of OW 01212016 M2 bacteria alone time course.wiff (Turbo Spra...
Max. 9.4e6 cps.
Intensity, cps
m/z, Da
199
359
279
297
327
341
227
217
261
141
145
163
173
111
119
135
137
187
201
209
243
245
253
277
287
299
315
323
360

Source Data

Donor 6

File name: OW 03162016 M1 M2 time polarization E coli RvD5 LTB4.wiff Samples (STD) and (OW M2 24 ecoli)

Donor 6: RvD2 and RvD5 **standard** chromatograms: OW 03162016 M1 M2 time polarization E coli RvD5 LTB4.wiff (STD)

Source Data

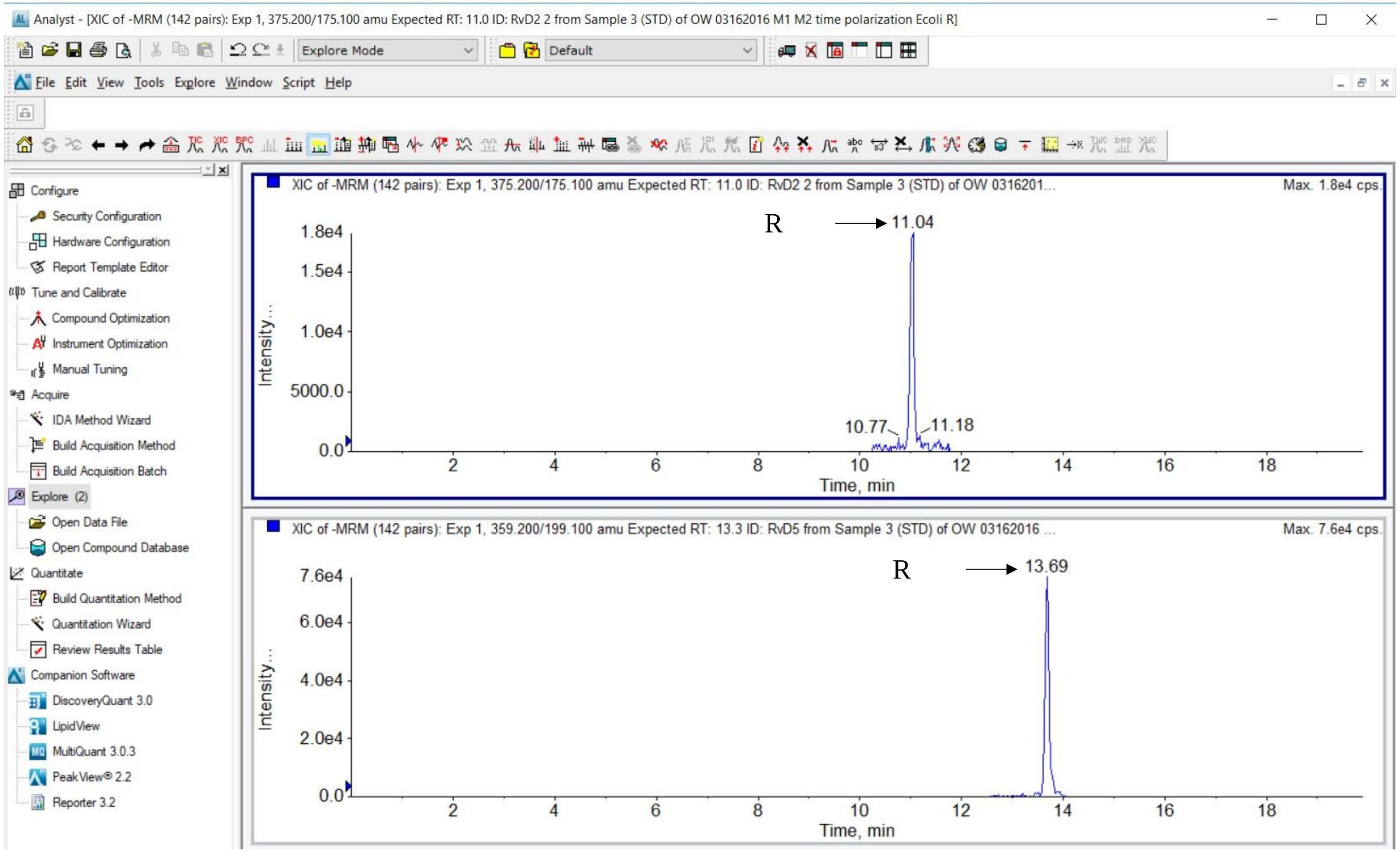

Donor 6: RvD2 and RvD5 chromatograms: OW 03162016 M1 M2 time polarization E coli RvD5 LTB4.wiff (OW M2 24 ecoli)

Source Data

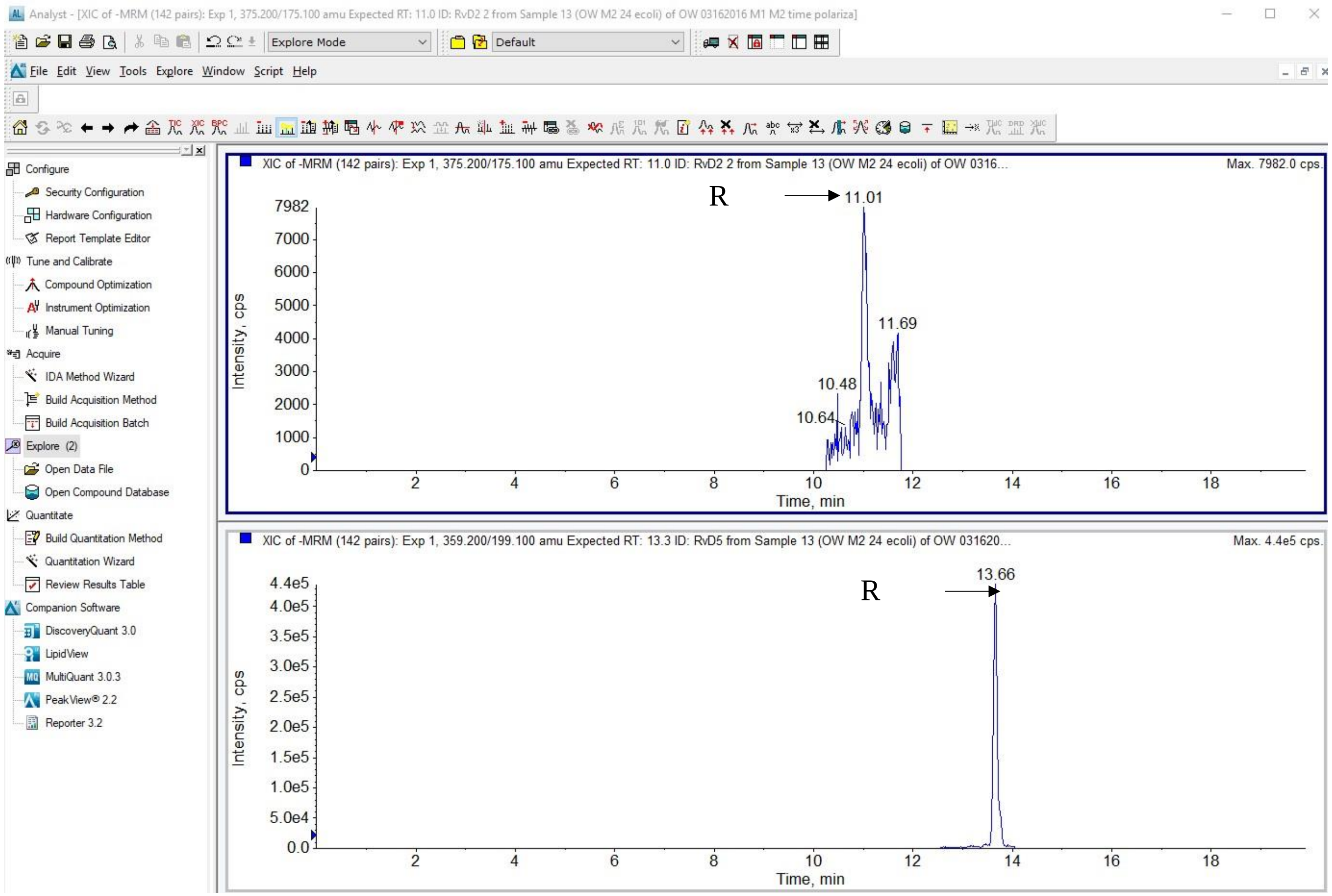

Donor 6: RvD2 EPI spectrum: OW 03162016 M1 M2 time polarization E coli RvD5 LTB4.wiff (OW M2 24 ecoli)

Source Data

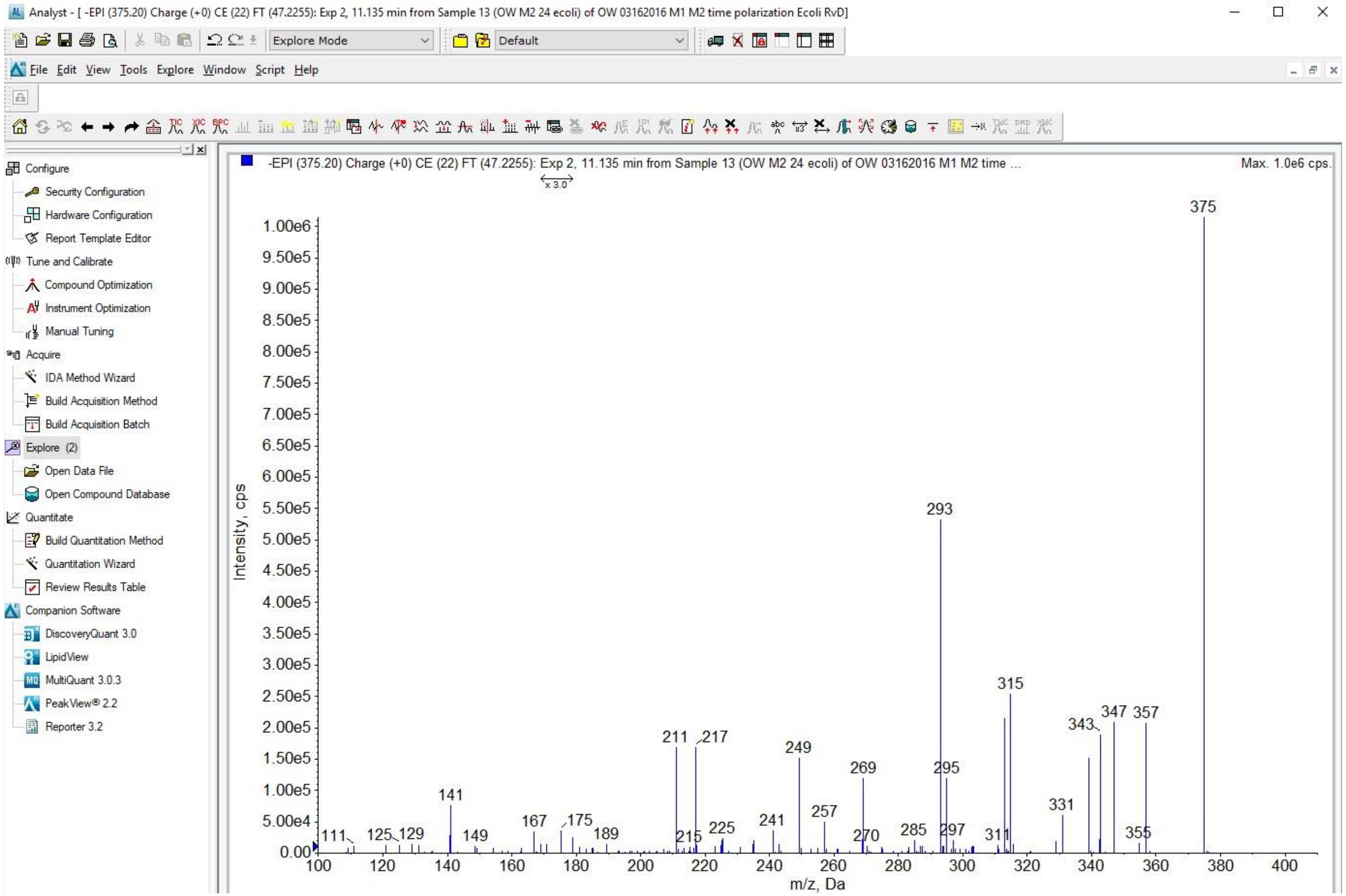

Donor 6: RvD5 EPI spectrum: OW 03162016 M1 M2 time polarization E coli RvD5 LTB4.wiff (OW M2-6)

Source Data

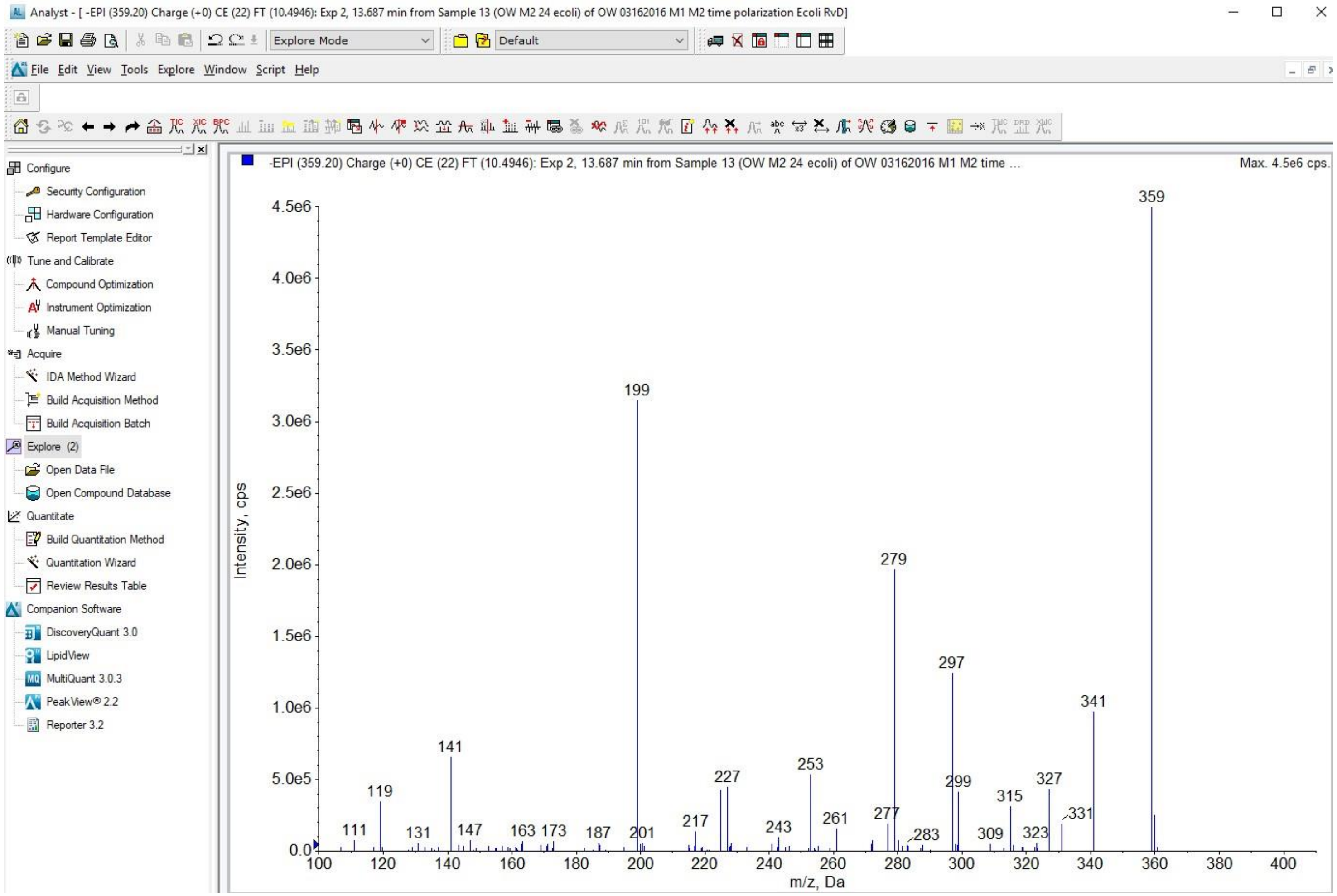

Analyst - [-EPI (359.20) Charge (+0) CE (22) FT (10.4946): Exp 2, 13.687 min from Sample 13 (OW M2 24 ecoli) of OW 03162016 M1 M2 time polarization Ecoli RvD]
Explore Mode
Default
File Edit View Tools Explore Window Script Help
Configure
Security Configuration
Hardware Configuration
Report Template Editor
Tune and Calibrate
Compound Optimization
Instrument Optimization
Manual Tuning
Acquire
IDA Method Wizard
Build Acquisition Method
Build Acquisition Batch
Explore (2)
Open Data File
Open Compound Database
Quantitate
Build Quantitation Method
Quantitation Wizard
Review Results Table
Companion Software
DiscoveryQuant 3.0
LipidView
MultiQuant 3.0.3
PeakView® 2.2
Reporter 3.2
-EPI (359.20) Charge (+0) CE (22) FT (10.4946): Exp 2, 13.687 min from Sample 13 (OW M2 24 ecoli) of OW 03162016 M1 M2 time ...
Max. 4.5e6 cps.
Intensity, cps
m/z, Da
359
199
279
297
341
141
119
227
253
299
315
327
331
111
131
147
163
173
187
201
217
243
261
277
283
309
323

Source Data

Donor 7

OW 03162016 M1 M2 time polarization E coli RvD5 LTB4.wiff

Samples (STD) and (OW M2-6)

Donor 7: RvD2 and RvD5 **standard** chromatograms: OW 03162016 M1 M2 time polarization E coli RvD5 LTB4.wiff (STD)

Source Data

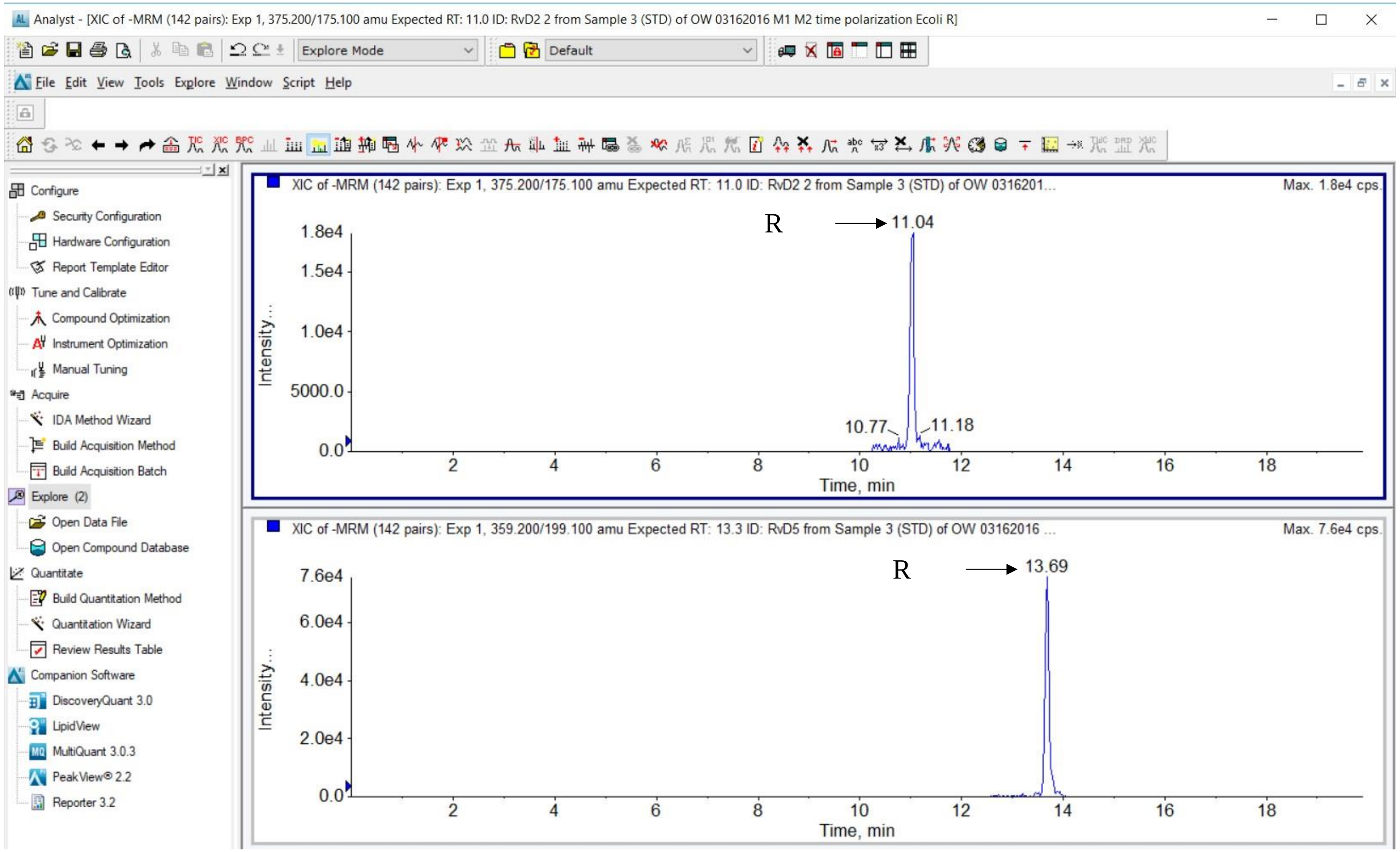

Donor 7: RvD2 and RvD5 chromatograms: OW 03162016 M1 M2 time polarization E coli RvD5 LTB4.wiff (OW M2-6)

Source Data

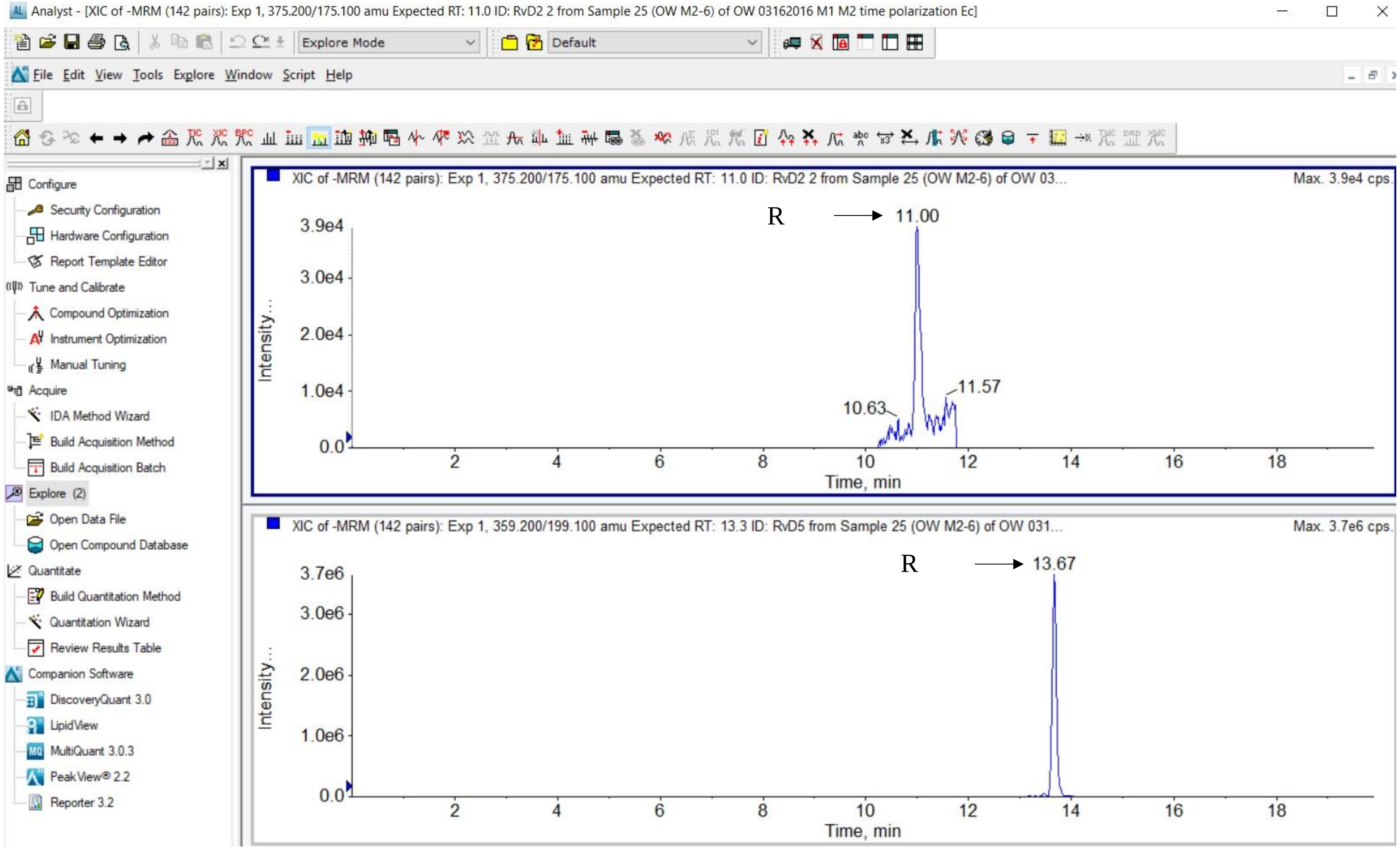

Donor 7: RvD2 EPI spectrum: OW 03162016 M1 M2 time polarization E coli RvD5 LTB4.wiff (OW M2-6)

Source Data

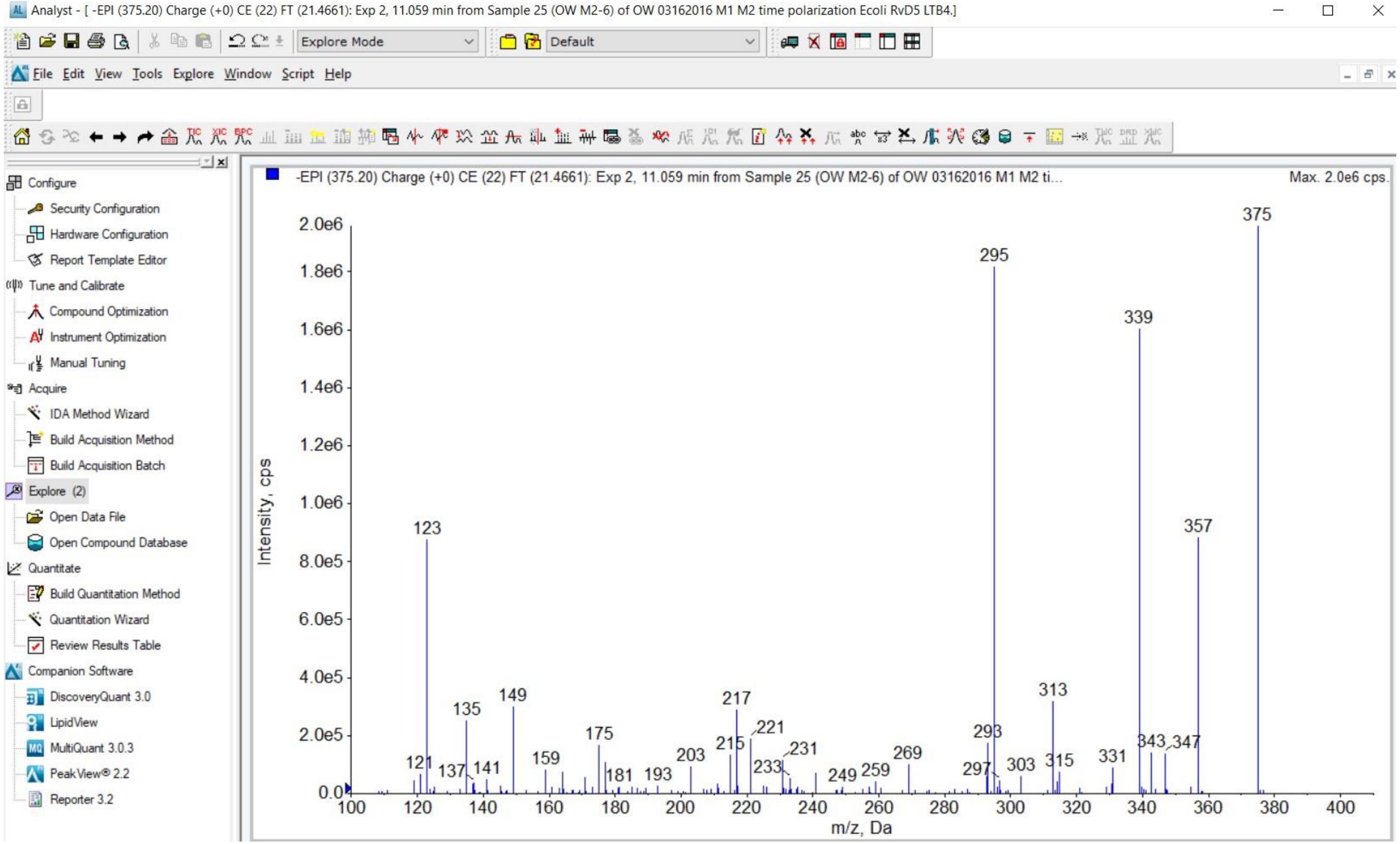

Donor 7: RvD5 EPI spectrum: OW 03162016 M1 M2 time polarization E coli RvD5 LTB4.wiff (OW M2-6)

Source Data

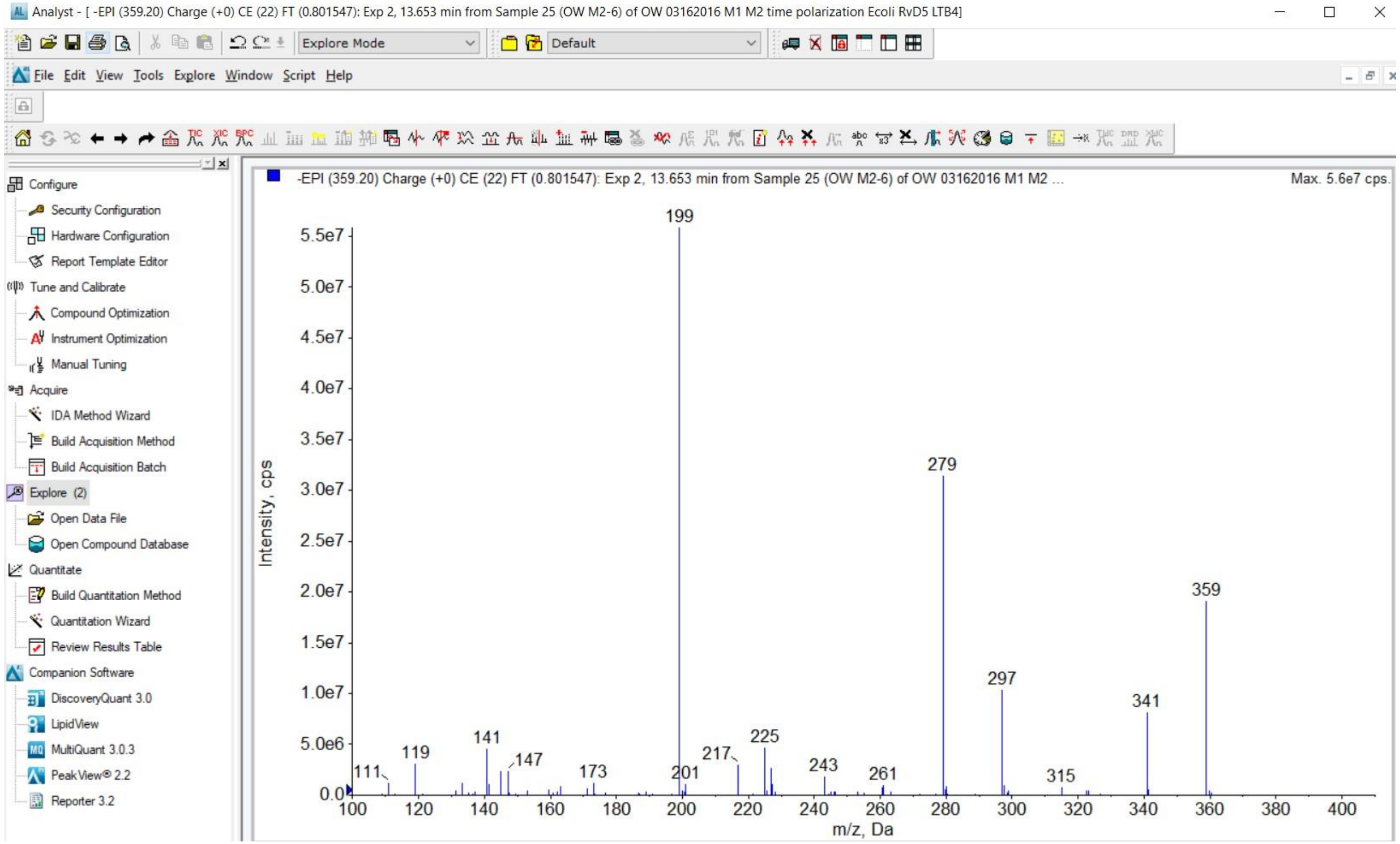

Analyst - [-EPI (359.20) Charge (+0) CE (22) FT (0.801547): Exp 2, 13.653 min from Sample 25 (OW M2-6) of OW 03162016 M1 M2 time polarization Ecoli RvD5 LTB4]
Explore Mode
Default
File Edit View Tools Explore Window Script Help
Configure
Security Configuration
Hardware Configuration
Report Template Editor
Tune and Calibrate
Compound Optimization
Instrument Optimization
Manual Tuning
Acquire
IDA Method Wizard
Build Acquisition Method
Build Acquisition Batch
Explore (2)
Open Data File
Open Compound Database
Quantitate
Build Quantitation Method
Quantitation Wizard
Review Results Table
Companion Software
DiscoveryQuant 3.0
LipidView
MultiQuant 3.0.3
PeakView® 2.2
Reporter 3.2
-EPI (359.20) Charge (+0) CE (22) FT (0.801547): Exp 2, 13.653 min from Sample 25 (OW M2-6) of OW 03162016 M1 M2 ...
Max. 5.6e7 cps.
Intensity, cps
5.5e7
5.0e7
4.5e7
4.0e7
3.5e7
3.0e7
2.5e7
2.0e7
1.5e7
1.0e7
5.0e6
0.0
199
279
359
297
341
141
119
111
147
173
201
217
225
243
261
315
100
120
140
160
180
200
220
240
260
280
300
320
340
360
380
400
m/z, Da

Source Data
